# Fluorescent Carbon Dots from Biomass Waste: Photoluminescent Behavior and Toxicity Profile Validated *In Vitro* and *In Vivo*

**DOI:** 10.1021/acsomega.5c12878

**Published:** 2026-05-22

**Authors:** Luana Caroline de Oliveira Lima, Janaina Domingas Alves, Amanda Maria Siqueira Moreira, Erika Cristina Jorge, Hélio Batista dos Santos, Ralph Gruppi Thomé, Paulo Henrique Almeida Campos-Junior, Marco Antônio Schiavon

**Affiliations:** † Grupo de Pesquisa em Química de Materiais (GPQM), Departamento de Ciências Naturais, 573437 Universidade Federal de São João del-Rei, São João del-Rei 36.301-160, Brazil; ‡ Laboratório de Pesquisa em Reprodução (LAPER), Departamento de Ciências Naturais, Universidade Federal de São João del-Rei, São João del-Rei 36.301-160, Brazil; § Laboratório de Biologia Oral e do Desenvolvimento (LABODE), Departamento de Morfologia, 28114 Universidade Federal de Minas Gerais, Belo Horizonte 31.270-901, Brazil,; ∥ Laboratório de Processamento de Tecidos, Campus Centro-Oeste Dona Lindu, Universidade Federal de São João del-Rei, Divinópolis 35.501-296, Brazil

## Abstract

Carbon dots (CDs) are emerging nanomaterials with tunable optical properties and promising scalability; however, their potential toxicity remains a key concern for biomedical and environmental applications. In this study, we investigated the *in vitro* and *in vivo* toxicity of CDs synthesized from spent coffee grounds (SCG-CD) via pyrolysis. Thermogravimetric analysis (TGA) was used to determine the optimal synthesis parameters, while fluorescence spectroscopy was used to confirm CD formation and excitation-dependent emission. The Design of Experiments (DoE) approach identified conditions yielding blue-emissive CDs with a maximum photoluminescence quantum yield of 6% (400 °C, 3 h, named S4_SCG-CD). Fluorescence analysis revealed pH-dependent emission enhancement under alkaline conditions. Structural characterization by Fourier-transform infrared spectroscopy and X-ray diffraction demonstrated surface functionalization and a semicrystalline framework. *In vitro* MTT assays indicated no cytotoxicity across tested concentrations, and *in vivo* assays in mice and zebrafish embryos revealed no acute toxicity. The S4_SCG-CD sample was classified as Category 5 (LD50 ≥ 5000 mg/kg) under the Globally Harmonized System and “practically nontoxic” (LC50 > 150 μg/mL) according to the Fish and Wildlife Service. These findings highlight spent coffee grounds as a sustainable precursor for generating biocompatible, low-toxicity materials with potential applications in bioimaging and eco-friendly nanotechnology.

## Graphical abstract

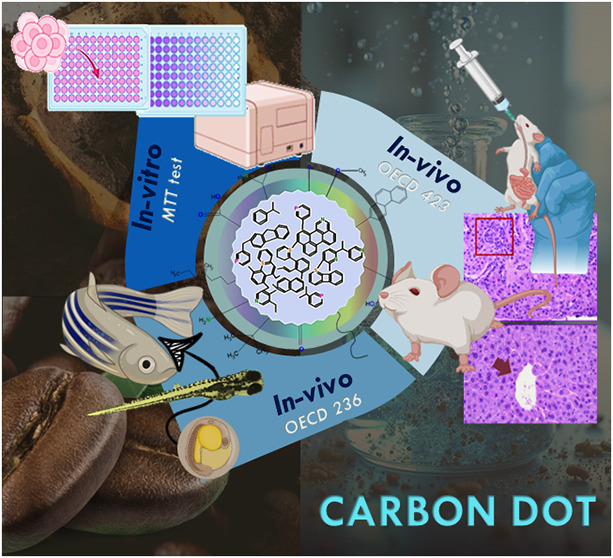

## 1 Introduction

Carbon dots (CDs) have emerged as a prominent class of carbon-based nanomaterials, attracting increasing interest due to their photoluminescent properties, facile synthesis, high water dispersibility, chemical stability, and low-cost precursors.^1^ Since their discovery in 2004,^2^ CDs have been extensively explored for applications in bioimaging,^3^ sensing,^4^ environmental remediation,^5^ nanomedicine,^6^ and optoelectronics, such as luminescent solar concentrators (LSCs)^7^ and electroluminescent white light-emitting diodes (WLEDs).^8^ Their tunable optical properties can be explained by different approaches: quantum confinement,^5,9^ surface states,^10,11^ and surface functionalities.^12,13^ Their versatility in those broad fields is guaranteed by their synthetic methodology, precursor availability, and optical properties.

While a wide range of synthesis procedures, covering both top-down and bottom-up strategies, have been developed and refined, the use of biomass-derived precursors has gained significant attention due to its alignment with green chemistry principles and the circular economy.^14–16^ Among the most promising biomass sources, spent coffee grounds (SCG) stand out for their abundance, low cost, and carbon-rich composition.^17–19^ However, despite the growing interest in biomass-derived CDs, comprehensive assessments of their biological and environmental safety remain scarce.^9,20^

Although extensive studies have reported the biocompatibility and environmental benignity of CDs, their toxicity has been shown to depend on multiple factors, such as synthesis routes, purification methods, and exposure conditions.^21,22^ Furthermore, the physicochemical characteristics of CDs, including particle size, surface charge, and functional groups, play a key role in cellular interactions and biodistribution.^23,24^ Supjaroenpisan et al.^25^ reported promising results for CDs derived from kitchen sugar doped with N, P, and S. The interaction of nondoped and phosphorus-doped carbon dots with HeLa cells resulted in low cytotoxicity, with cell viability exceeding 95% at a concentration of 1 g·L^–1^ after 24 h of incubation.

However, toxicological thresholds (e.g., LD_50_ and LC_50_) for CDs synthesized from identical precursors via different methods remain largely unexplored, highlighting the need for standardized protocols and multimodel assessments to elucidate dose-dependent effects, bioaccumulation, and systemic impacts.^26^ Lin et al. evaluated lysine-functionalized carbon dots (Lis-CDs) using zebrafish and three mammalian models (rabbits, guinea pigs, and rats). While embryonic and eleuthero-embryonic zebrafish showed severe toxic effects at concentrations 15 times higher than the authorized dose (0.5 ppm), adult zebrafish exhibited no adverse effects under prolonged exposure. Even at 2000 mg/kg, Lis-CDs cause no weight loss, skin irritation, or sensitization in mammals. These results indicate that the toxicity of Lis-CDs is development-stage-dependent in zebrafish and differs from mammalian responses, emphasizing the importance of carefully evaluating each toxicological profile in comparative studies. This underscores the need for standardized protocols and multimodel biological assessments to elucidate dose-dependent effects, bioaccumulation, and systemic impacts to ensure their safe deployment in technological applications.

In line with this need for harmonized evaluation strategies, Bi and co-workers^27^ have conducted a recent study proposing an integrated and standardized approach for rapid biotoxicity screening of CDs combining *in vitro* and *in vivo* models. A systematic framework incorporating complementary cytotoxicity assays (such as metabolic activity, membrane integrity, and flow cytometry-based analyses) together with zebrafish embryotoxicity evaluation—monitoring end points including hatching rate, morphological alterations, and developmental progression—has been suggested as a robust strategy to improve reproducibility and comparability across studies. Such multiend point platforms provide a more comprehensive understanding of dose-dependent biological responses and reinforce the importance of unified toxicological assessment criteria before biomedical or environmental implementation of carbon-based nanomaterials.

In this context, increasing attention has been paid to the environmental impact of CDs, particularly in aquatic systems, which has added an additional pathway toward the development of safer nanomaterials.^20,28–32^ In this context, zebrafish (*Danio rerio*), widely recognized as a model organism for toxicological assessment, have been used to assess acute and developmental toxicity.^33,34^ These *in vivo* models provide a bridge between *in vitro* findings and ecological relevance and can be directed toward analytical applications, such as portable selective ion sensors, which offer rapid and easy detection. For instance, Costa and colleagues evaluated the toxicity of carbon dots produced via hydrothermal carbonization of the mesocarp of the babassu coconut (Orbignya speciosa). Following the observation of negligible toxicity, the carbon dots were employed in fluorescence quenching assays in the presence of common ions in aquatic matrices (Fe^3+^, Co^2+^, Ni^2+^, Zn^2+^, Cu^2+^, and Cd^2+^), showing good linearity in fluorescence decay and demonstrating their potential as environmental sensors.

In this context, we report the synthesis of fluorescent CDs from spent coffee grounds via pyrolysis under oxidizing air conditions. A design of experiments approach was applied to identify optimal synthesis conditions. DL-tartaric acid (TA) was used as a comparative precursor because of its simple, well-defined, and oxygen-rich organic molecule composed exclusively of C, H, and O, employing a chemically simple and structurally controlled reference for the synthesis and characterization of the CDs. The optical and structural characterizations were performed. The cytotoxicity of the optimized spent coffee ground carbon dots was evaluated using MTT assays, and acute toxicity was assessed *in vivo* using zebrafish and mice as complementary biological models. This study provides a comprehensive analysis of their physicochemical properties and biosafety profiles.

## 2 Experimental Section

### 2.1 Materials and Reagents

The spent coffee grounds were obtained from homemade filtered coffee. The material was dried in an oven at 105 °C for 24 h before use. Ultrapure water (Milli-Q Direct 8/16, Millipore) was used in all aqueous solutions. Acetic acid (99.7%, Synth), boric acid (ACS grade), DL-tartaric acid (99%, Sigma-Aldrich), sulfuric acid (95–99%, Vetec), ethanol (70°, Itajá), methylene blue (Sigma-Aldrich), calcium chloride (Êxodo Científica), potassium chloride (Synth), sodium chloride (ACS grade), sodium phosphate monobasic dihydrate (99%, Synth), sodium phosphate dibasic anhydrous (98%, Synth), sodium hydroxide (98%, Synth), paraformaldehyde (Neon), paraplast (Sigma-Aldrich), magnesium sulfate (Êxodo Científica), and quinine sulfate (99%, Sigma-Aldrich) were used as received without further purification.

### 2.2 Samples Screening

#### 2.2.1 Thermogravimetric Analysis

A thermogravimetric analysis was performed to investigate the thermal profiles of DL-tartaric Acid (TA) and spent coffee grounds (SCG) to assess the study levels of each factor (temperature and time) to be used in the DoE. The influence of the heating rate (β) of 5, 10, and 15 °C·min^–1^ on the thermal decomposition profile was also evaluated. All samples were analyzed using the Shimadzu TGA/DTA-60H system, with heating from 25 to 700 °C under synthetic air flow (50 mL/min).

##### 2.2.1.1 Carbon Dot Core Formation Study by Photoluminescence

A photoluminescence emission study was conducted as a function of the synthesis temperature, guided by the exploratory thermogravimetric analysis described in Section 2.2.1. Samples were prepared by dispersing the total mass obtained after purification in 3 mL of ultrapure water for each synthesis condition; therefore, no fixed concentration was employed for the spectral acquisition. Emission spectra were recorded by using an RF-5301PC spectrofluorometer (Shimadzu) equipped with a 150 W xenon lamp as the excitation source. Both excitation and emission slits were set to 3, and all measurements were conducted at room temperature in quartz cuvettes with a path length of 1 cm.

#### 2.2.2 Design of Experiments

A full factorial design with three levels (−1, 0, 1), two factors (temperature and time), and triplicate measurements was employed to optimize the synthesis, resulting in *N* = *n*.3^k^ equation, where N denotes the total number of experiments, *n* the number of experimental replicates, and k the number of factors investigated. The equation resulted in 27 experimental runs, which were coded and arranged in a randomized order to avoid systematic errors. The average relative photoluminescence intensity was the response variable in the Minitab.

### 2.3 Synthesis and Purification of Carbon Dots

CDs were synthesized from spent coffee grounds following a procedure adapted from previous reports.^35–37^ Spent coffee grounds were dried at 105 °C for 24 h prior to use. DL-tartaric acid was employed as a comparative precursor. Both CDs were synthesized using 2.50 g and pyrolyzed in an alumina tubular furnace (EDG 10P–S) under oxidizing atmosphere, at 10 °C·min^–1^ heating rate, at the temperature and time set by the DoE. The crude dark-brown product was macerated, dispersed in 50 mL of ultrapure water, and sonicated (40 kHz, 30 min, Ultronique). The mixture was subsequently heated to 100 °C for 1 h under stirring, centrifuged at 5400 rpm for 30 min (Hettich), and filtered through a PVDF membrane (0.22 μm pore size, Micropore). The filtrate was concentrated using a rotary evaporator (Laborota 4000 efficient, Heidolph) under vacuum at 60 °C and dried in a circulating air oven (Equilam) at 105 °C for 12 h. Dried samples were dispersed in 3 mL of ultrapure water and immediately characterized.

### 2.4 Characterization of Optimized CDs

#### 2.4.1 DoE: Carbon Dot Core Formation Study by Optical and Statistical Analysis

UV–vis absorbance spectra were recorded from 200 to 600 nm using a Shimadzu UV-2550 spectrophotometer with 10 mm quartz cuvettes. PL was measured as described in 2.2.1.1. The photoluminescence quantum yield (PLQY) was calculated using the comparative method proposed by Williams, employing quinine sulfate in ultrapure water as a standard; linear fitting of integrated emission intensity versus absorbance was applied (see eq 1 in Supporting Information). Time-resolved photoluminescence (PL) decay measurements were conducted using a Horiba Fluorolog-3 Jobin-Yvon system with a nanoLED pulsed source at 340 nm. Functional groups were identified by attenuated total reflectance Fourier-transform infrared (ATR-FTIR) spectroscopy (Bruker Vertex 70 & Hyperion 3000) over 400–4000 cm^–1^. The crystallographic properties were analyzed via X-ray diffraction (XRD) using a Shimadzu diffractometer with Cu Kα radiation (λ = 1.5418 Å) at 30 kV and 30 mA. Morphology and elemental composition were examined by scanning electron microscopy (SEM) on a HITACHI TM3000, coupled with energy-dispersive spectroscopy (EDS) using a QUANTAX70 detector.

#### 2.4.2 Study of the Dependence of Photoluminescence on pH Variations of SCG-CD

The influence of pH on the photoluminescence of SCG-CD was determined using Britton–Robinson buffer (pH 3–11). The buffer solution was prepared by mixing 0.10 M solutions of acetic acid, phosphoric, and boric acids. The pH was adjusted using 2.0 M NaOH. The emission spectra were recorded following the procedure described in topic 2.2.1.1, using an excitation wavelength of 310 nm.

### 2.5 Toxicity Evaluation

#### 2.5.1 *In Vitro* Toxicity Testing of S4_SCG-CD Sample

Cell viability was assessed using the MTT assay. Briefly, 1 × 10^4^ 3T3 fibroblasts were seeded into 24-well plates and exposed to S4_SCG-CD at concentrations of 5, 10, 20, 40, 80, and 160 μg/L for 48 h. The culture medium was replaced with MTT solution (Invitrogen) after exposure, and the samples were incubated for 4 h at 37 °C under a 5% CO_2_ atmosphere. The resulting formazan crystals were dissolved in 1 mL of acidified isopropanol. Subsequently, the solutions were transferred to a 96-well plate in triplicate, and the absorbance was measured at 595 nm using a microplate reader (ELx800, BioTek). This assay evaluates cell viability based on mitochondrial enzymatic activity, which reduces MTT to insoluble formazan crystals, indicating metabolically active cells.

#### 2.5.2 *In Vivo* Toxicity Testing of the S4_SCG-CD Sample in Mice

Acute oral toxicity tests were performed according to the OECD Guidelines 423.^38^ Mice were divided into control and test groups and administered 300 and 2000 mg/kg of S4_SCG-CD. Behavioral changes were monitored throughout the study. After euthanasia, biochemical assays were conducted to assess potential internal damage or infection. Mice were obtained from the Laboratory Animal Breeding Center (NUCAL) at the Federal University of São João del-Rei (UFSJ), under protocol number 5020041223, approved by the Ethics Committee on Animal Use of the Federal University of São João del-Rei (CEUA/UFSJ).

#### 2.5.3 Toxicity Testing in Zebrafish Embryos of the S4_SCG-CD Sample

The Fish Embryo Acute Toxicity (FET) test was conducted following OECD Guideline 236.^33^ The study was approved by CEUA/UFSJ under protocol number 9671011222. Zebrafish embryos were exposed to S4_SCG-CD at concentrations of 150, 200, 250, and 500 μg/L for 96 h postfertilization (hpf). The primary end points were survival percentage (defined by embryonic coagulation and absence of heartbeat) and hatching rate, with the presence of malformations recorded. Statistical analysis was performed using analysis of variance (ANOVA) followed by Tukey’s post hoc test, with *p* < 0.05 considered statistically significant. GraphPad Instat (version 3.5) was used for all analyses.

## 3 Results and Discussion

### 3.1 Samples Screening

#### 3.1.1 Thermogravimetric Analysis

The thermal decomposition profiles of both precursors were investigated. Both samples exhibit a systematic shift with decomposition peaks toward higher temperatures by increasing β, which is attributed to reduced heat transfer efficiency and altered gas–solid interactions (see Supporting Information, Figure S1a,d).^39^

The optimal thermal profile was observed at a heating rate of 10 °C/min by derivative thermogravimetry (DTG) (Figure S1b–e), which clearly distinguished the secondary thermal events at this rate. For TA, these events occurred between ∼350 and 450 °C, and for SCG between ∼400 and 550 °C. They likely arise from processes such as dehydration, oxidation, and degradation of organic acids in both precursors (Figure S1c–f).

At β = 10 °C/min, TA (Figure 1a) showed initial mass loss from water release (23 to 160 °C),^36^ fusion onset at 196 °C,^40^ and major decomposition at 238 °C corresponding to pyruvate breakdown,^41^ and a secondary peak at 402 °C, indicating degradation of residual acids.^41^ In contrast, SCG (Figure 1b) displayed broader events, including dehydration (30 to 127 °C),^42^ active pyrolysis (300 to 400 °C),^43^ and passive pyrolysis (400 to 500 °C), consistent with the degradation of hemicellulose, cellulose, and lignin.^44,45^ Thus, the carbonization windows were defined as 260 to 400 °C for TA and 280 to 400 °C for SCG.

**Figure 1:**
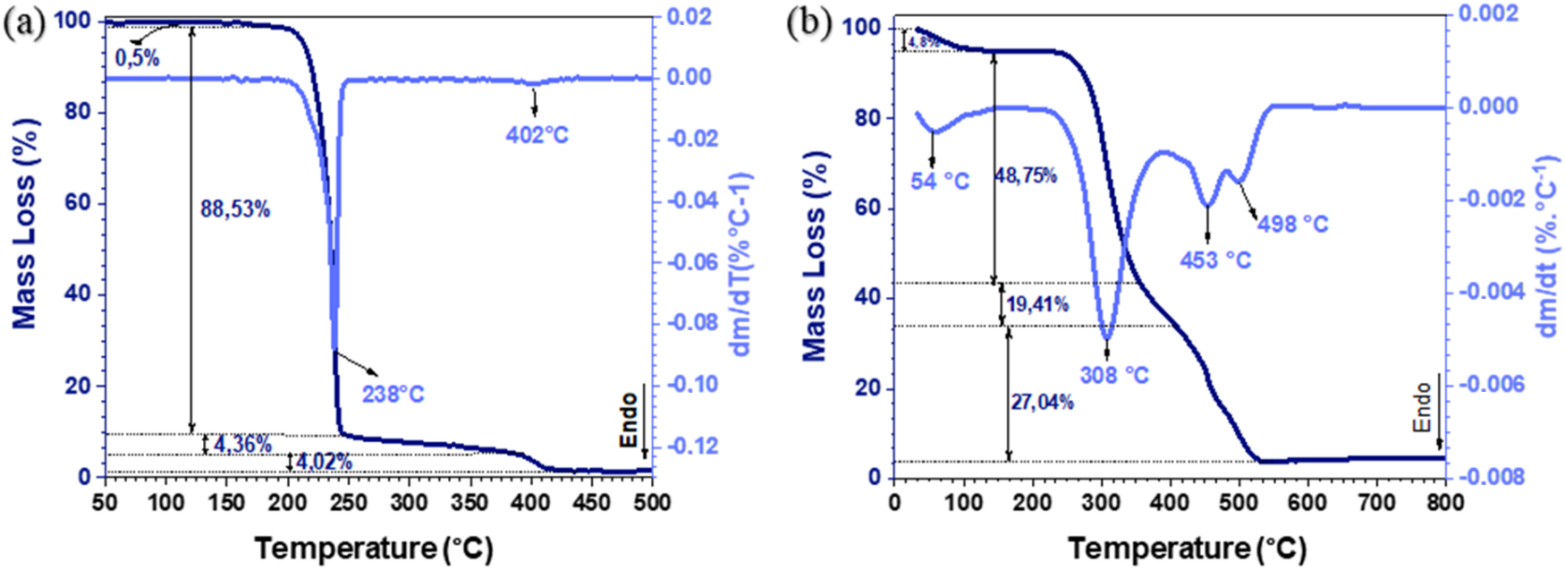
TGA and DTG thermograms: (a) tartaric acid and (b) spent coffee ground, β = 10 °C/min, under a synthetic air atmosphere.

The mass loss percentage and amount of residual material in the thermogram were considered to guide further characterizations and applications. Taking TA as a standard, the maximum working temperature exhibited by TGA was 400 °C. Therefore, isothermal experiments were conducted for 1 h, 2 h, and 3h at this temperature. At 400 °C and 3 h, TA decomposed faster, likely due to its simpler molecular structure, whereas SCG exhibited roughly twice the residual mass. Time-dependent isotherms provided yields of 0.48% (m/m) for TA and 0.844% (m/m) for SCG after 3 h (Figure S2a,b).

### 3.2 Carbon Dot Core Formation Study by Photoluminescence

After identifying the maximum temperature of the carbonization windows, it was necessary to verify the temperatures at which the process should be started and whether it led to proper carbon core formation. Incomplete or poor core formation can compromise the final material quality. To this end, the PL was evaluated. For TA-CD synthesis, temperatures between 260 and 280 °C yielded negligible PL due to incomplete and nonhomogeneous carbonization, whereas 300 to 400 °C for 3 h produced samples with visually uniform carbonization and stable PL (Figure S3a–d). In contrast, SCG-CDs exhibited consistent PL within the effective temperature range of 300–400 °C (Figure S4a–d). These results guided the selection of 300 °C, 350 °C, and 400 °C as synthesis temperatures.

### 3.3 Characterization of Optimized CDs

#### 3.3.1 DoE: Carbon Dot Core Study Formation by Optical and Statistical Analysis

The factorial design was employed to optimize CDs synthesis from TA and SCG, using temperature at 300 °C, 350 °C, 400 °C, and time at 1 h, 2 h, 3 h as variables (Figure S5). An experimental design with three levels enables a deeper assessment of factor interactions and how they affect the outcome or response variable. It is well-suited for complex systems, such as SCG-CDs, and for obtaining optimized conditions, whether aiming to maximize or minimize an undesired response. In our study, the optimized condition aimed for a high photoluminescence efficiency, which was assessed via the average relative intensity of photoluminescence, which, under certain conditions, may be equivalent to photoluminescence quantum yield (PLQY).

By considering only the photoluminescence emission spectral profiles, it is possible to infer the influence of the synthesis parameters on PL behaviors (Figures S5–S8). For both TA-CDs (Figures S5–S6) and SCG-CDs (Figures S6–S8), a clear enhancement in fluorescence intensity was observed as a function of increasing synthesis temperature and reaction time, respectively. TA-CDs exhibit more uniform spectral profiles, with emission maxima located in closely related regions, whereas SCG-CDs display two distinct emission maxima depending on λ_exc_, indicating a higher degree of material heterogeneity.^10,11^ Both TA-CDs and SCG-CDs showed an excitation-dependent photoluminescence. TA-CDs exhibited maximum emission peaks using a λ_exc_ at 310 and 320 nm, and SCG-CDs exhibited maximum emission peaks using a λ_exc_ at 310, 320, 350, and 360 nm.

After this preliminary analysis, the data were input into Minitab by using the average relative fluorescence intensity as the response parameter. To this end, the same optical geometry, detection setup, and excitation wavelengths were selected. The absorbance at the excitation wavelength was used as a stand-in for the quantity of absorbed photons under low-absorption conditions, as covered in traditional and standardized PL measurements. This strategy enables a relative comparison of emission intensities. This method works well for comparative studies and trend analysis, which is the focus of the current work, even though it does not replace absolute PLQY measurements using an integrating sphere.^46^ This approach was adopted because the materials obtained after synthesis often exhibited a resin appearance, preventing the preparation of dispersions with well-defined concentration. Therefore, the average relative fluorescence intensity was obtained from the absorption and emission data under identical experimental conditions: namely, 3 mL of solvent (ultrapure water) and identical excitation and emission slit widths.

Minitab output indicated two promising conditions for TA-CDs (Figure S9) and four for SCG-CDs (Figure S10), at different excitation wavelengths (λ_exc_) and synthesis conditions. From a statistical standpoint, the interaction between the experimental variables was analyzed to determine the factors that most strongly influenced the synthesis outcomes.

For TA-CD, the statistical models (Figure 2a–c) indicated no significant influence of temperature or time on its PL intensity (*p* = 0.316 and *p* = 0.475 for temperature and time, respectively, Tables S2–S3; eq 3 in the Supporting Information). However, the interaction plot revealed a drop in luminescence at the highest temperature tested (400 °C) and prolonged durations (3 h). This behavior was experimentally confirmed by visual inspection. Samples heated for more than 2 h showed extensive thermal degradation, resulting in an insufficient amount of recoverable material and ash formation. This observation contrasts with the TGA results, which indicated a residual mass of 0.48 wt % under the most severe conditions (400 °C, 3 h). The surface plot reflected this trend, and the optimal synthesis condition was identified at 400 °C for 2 h.

**Figure 2:**
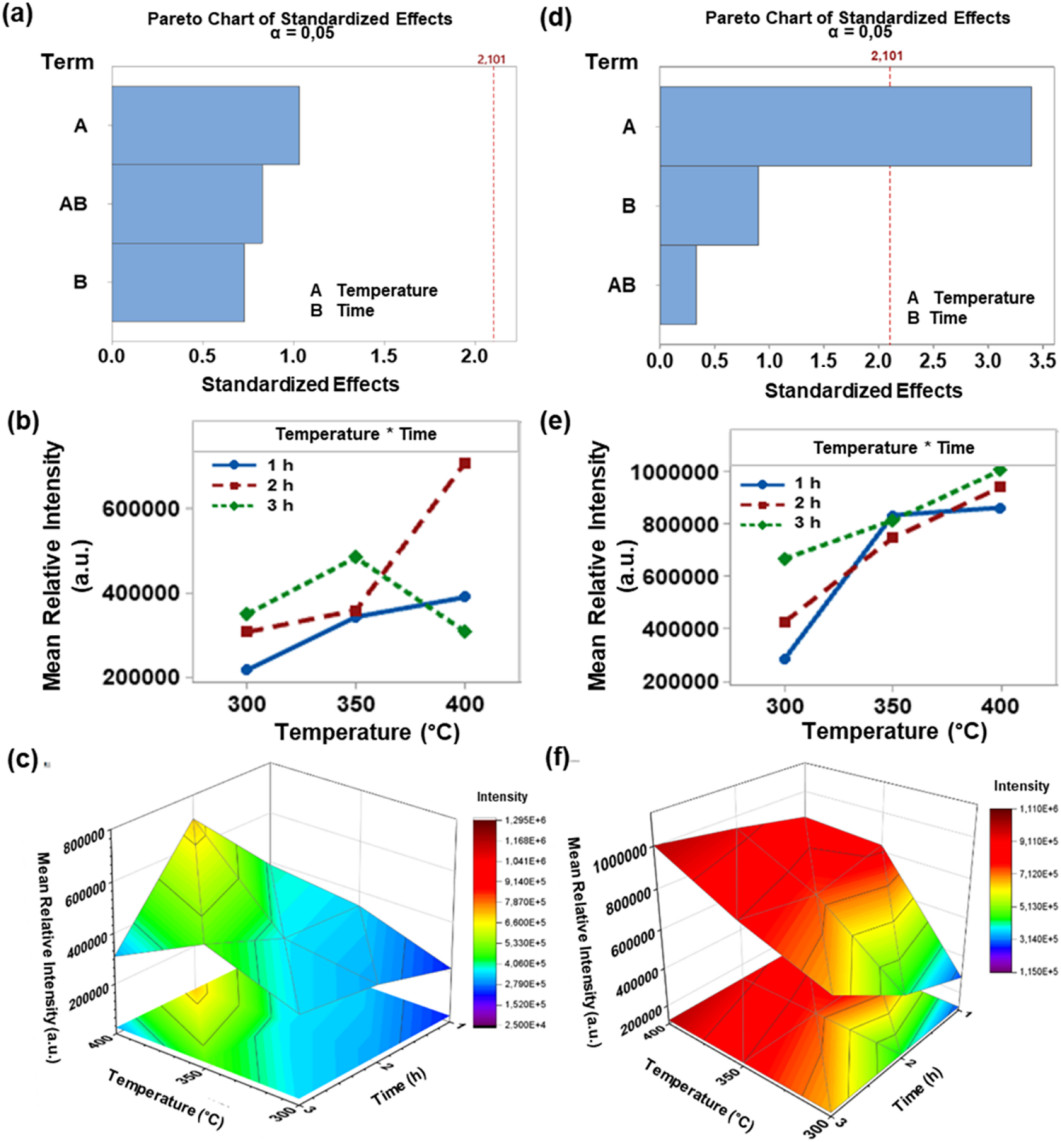
Statistical analysis of carbon dot synthesis. Pareto charts of standardized effects (a, d), interaction plots of temperature and time (b, e), and 3D surface maps of average relative photoluminescence intensity (c, f) for TA-CD (a–c) and SCG-CD (d–f) at an excitation wavelength of 310 nm.

SCG-CD showed a significant temperature dependence (Figure 2d–f), with PL intensity increasing at higher temperatures. Regression analysis confirmed temperature as the only statistically relevant factor (*p* = 0.010 and *p* = 0.381 for temperature and time, respectively, Tables S4–S5; eq 3 in the Supporting Information), and the surface plot identified at 400 °C for 3 h as the best condition.

To determine the excitation wavelength (λ_exc_) for subsequent characterizations and to validate the reliability of the DoE approach, the PLQY of these six selected samples was measured (Figures S9–S10). The PLQY results identified λ_exc_ = 310 nm as the optimal excitation wavelength for both precursors (Table S1). For TA-CDs, the optimal condition was 400 °C for 2 h, yielding a PLQY of 8.3%, and this sample was designated S9_TA-CD. The optimal condition for SCG-CDs was 400 °C for 3 h, resulting in a PLQY of 6.0%, designated S4_SCG-CD. These results are fully consistent with the conclusions obtained from the DoE, thereby confirming its effectiveness. Although the PLQY of SCG-CDs is 6.0%, this value remains suitable for bioimaging and biosensing applications, where biocompatibility, photostability, excitation tunability, and environmental responsiveness are often more critical than achieving maximum emission efficiency.

The DoE highlighted the critical role of temperature in CD synthesis and demonstrated that this statistical tool is effective for identifying optimal experimental conditions. Although the regression models provided useful insights, the DoE showed limited predictive accuracy in predicting future materials or synthesis outcomes. This result indicates that further refinement is required to improve the model’s robustness and predictive capability.

#### 3.3.2 Characterization of S9_TA-CD and S4_SCG-CD

The optical characterization of S9_TA-CD and S4_SCG-CD displays excitation-dependent photoluminescence (Figure S11), with two emissive centers visible in the surface maps (Figure 3a,b), a typical feature of carbon dots.^47,48^ S4_SCG-CD showed more intense signals at shorter wavelengths and a broader distribution of emissive surface states due to the heterogeneous precursor.^10,11^

**Figure 3:**
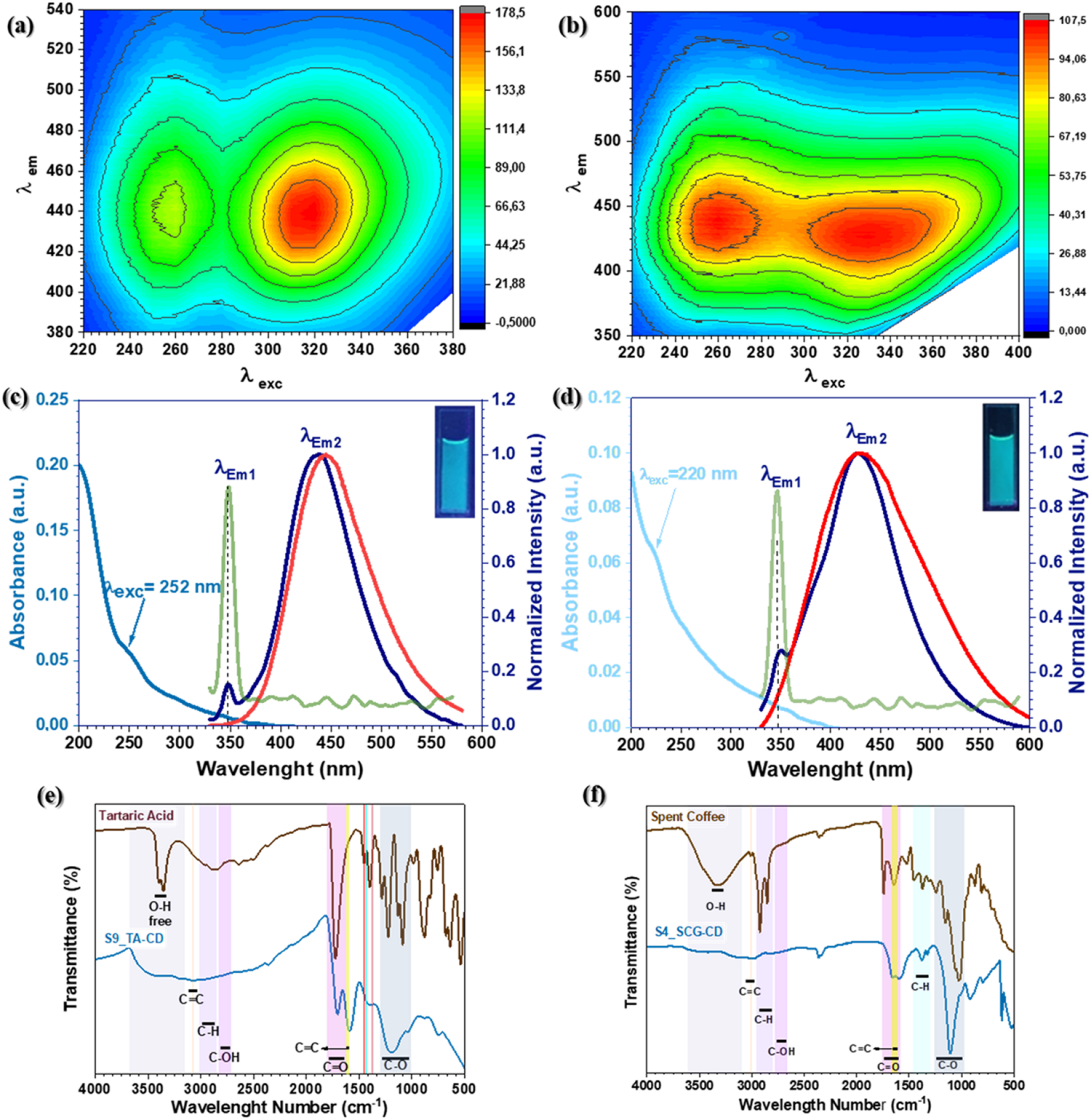
Excitation-wavelength-dependent fluorescence emission surface maps of S9_TA-CD (a) and S4_SCG-CD (b). UV–vis absorption and fluorescence emission spectra of S9_TA-CD (c), (400 °C, 2 h) and S4_SCG-CD (d), (400 °C, 3 h), including photographs under UV light. Color legend: absorption curves (c– S9_TA-CD, blue; d– S4_SCG-CD, light blue), water Raman emission (light green), sample emission (navy blue), and emission verified on a second instrument (red). Fourier-transform infrared spectra of TA and S9_TA-CD (e) and of spent coffee and S4_SCG-CD (f).

The UV–Vis spectra revealed π–π* transitions at 252 nm (S9_TA-CD) and 220 nm (S4_SCG-CD),^49^ corresponding to the aromatic domains (Figure 3c,d). The absence of n−π* transitions is attributed to oxygen-containing groups degradation during pyrolysis.^35^ Emission maxima were observed at 445 nm (S9_TA-CD) and 430 nm (S4_SCG-CD), Figure 3c,d, likely arising from the radiative recombination of surface carboxyl and hydroxyl groups.^49^ A minor peak at 350 nm, corresponding to Raman scattering from water, was confirmed via optical filtering.^50^

Time-resolved photoluminescence analysis (Figure S12, Table S6) revealed biexponential decay (see eq 4 in the Supporting Information), reflecting the coexistence of multiple emissive pathways.^12^ The short-lifetime component (τ_1_) was dominant, corresponding to direct exciton recombination, whereas the long-lifetime component (τ_2_) indicated trap-state-mediated decay. Consistent with its complex surface chemistry, S4_SCG-CD sample exhibited a higher average lifetime and greater delayed recombination.

FTIR spectroscopy showed oxidation-induced surface modifications, notably the conversion of hydroxyls into carbonyl-containing groups (Figure 3c–f, Tables S7–S8). S9_TA-CD formed alcohol, ketones, and conjugated aromatic domains, while S4_SCG-CD presented diverse functional groups (esters, amides, nitro compounds, amines, alkyl ethers, epoxides), enhancing hydrophilicity for aqueous applications.^51^

Additional features in the spectrum of S4_SCG-CD, particularly near 615 cm^–1^, were investigated using EDS and XRD (Figures S13–S14). EDS confirmed the presence of multiple elements (C, H, O, S, P, Na, Mg, Al, Cl, K, and Si), typical biomass-derived materials.^52^ The XRD patterns showed structural reorganization, with S4_SCG-CD displaying more defined peaks than its amorphous precursor, indicating the metal-driven ordering of carbon structures.

### 3.4 Study of the Response of Photoluminescence to pH Variations

The PL of S9_TA-CD and S4_SCG-CD were measured over pH 3 to 11, to assess their PL-behavior, mainly due to the biological tests (Figure 4). S9_TA-CD exhibited a modest blue shift (∼15 nm) and increased intensity at acidic pH (3 to 5), stabilization between pH 6 and 9, a maximum at pH 10, and decreased emission at pH 11 (Figure 4a,b).

**Figure 4:**
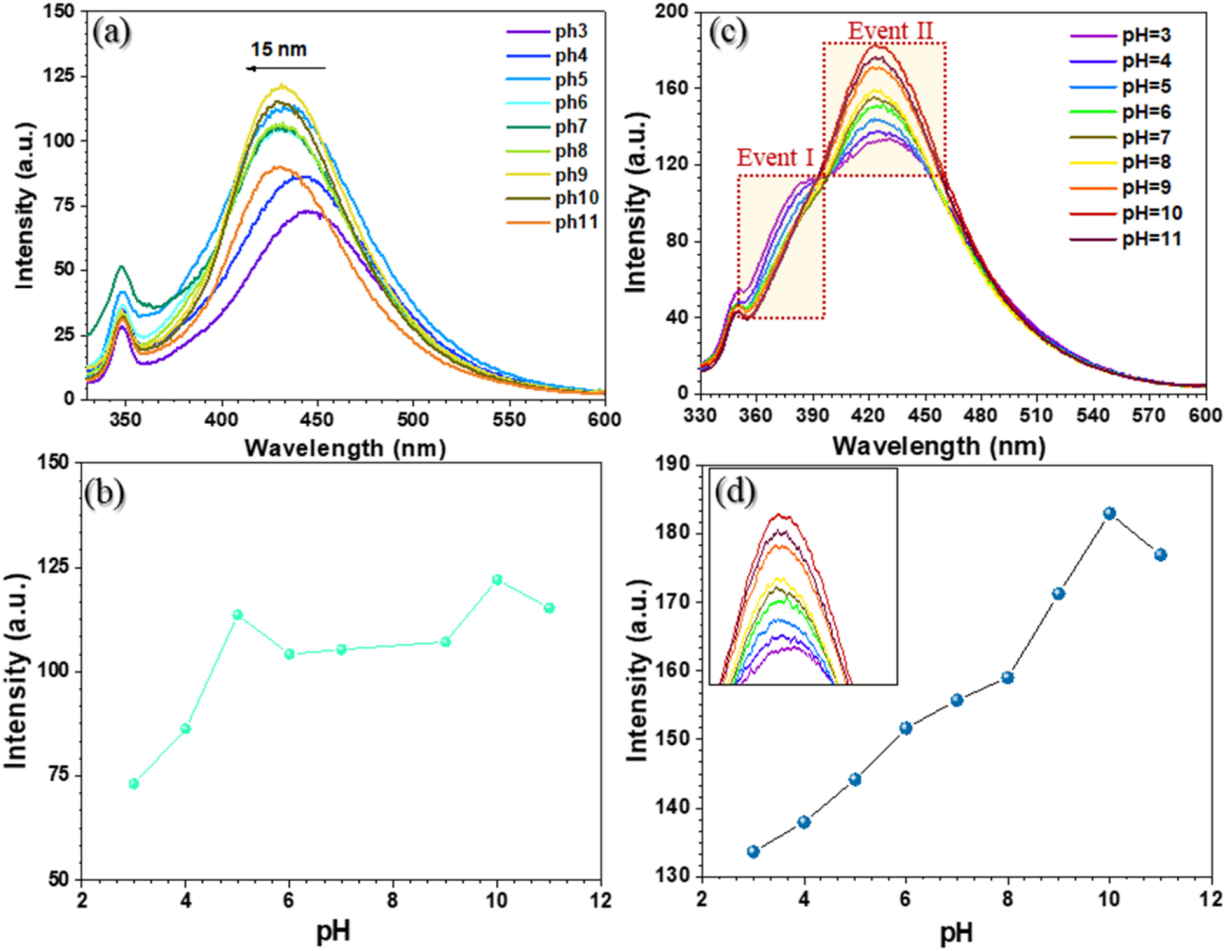
Photoluminescence response of carbon dots to variation in pH. Emission spectra of S9_TA-CD (a, b) and S4_SCG-CD (c, d) recorded at pH values from 3 to 11.

S4_SCG-CD showed a more pronounced pH response (Figure 4c,d), with increased PL intensity at higher pH and suppression of short-wavelength shoulders without spectral shift. Protonation of oxygenated groups under acidic conditions likely quenches emission by hindering n−π* transitions,^12,53^ while deprotonation under basic conditions enhances resonance in carboxyl and related groups, thereby increasing radiative recombination. This tunable PL highlights the potential of S4_SCG-CD’s for pH-responsive imaging and sensing applications in biologically relevant environments.^3–6^

### 3.5 Toxicity Assessment of the S4_SCG-CD Sample

#### 3.5.1 *In Vitro* Testing

3T3 fibroblast cells were exposed to S4_SCG-CD (5, 10, 20, 40, 80, and 160 μg/L) for 48 h.^54,55^ Cell viability remained unchanged at all concentrations (Figure 5a,b). A slight increase in proliferation was observed at 20 μg/L (*p* = 0.0209; control: 0.134 ± 0.0088; 20 μg/L: 0.1544 ± 0.0036, *n* = 7) which may be linked to the elemental composition of S4_SCG-CD (*e.g*., potassium), which is well established as a modulator of cellular activity. Previous studies have reported negligible cytotoxicity for similar CDs and graphene quantum dots, with surface chemistry playing a key role in biocompatibility.^17,56,57^

**Figure 5:**
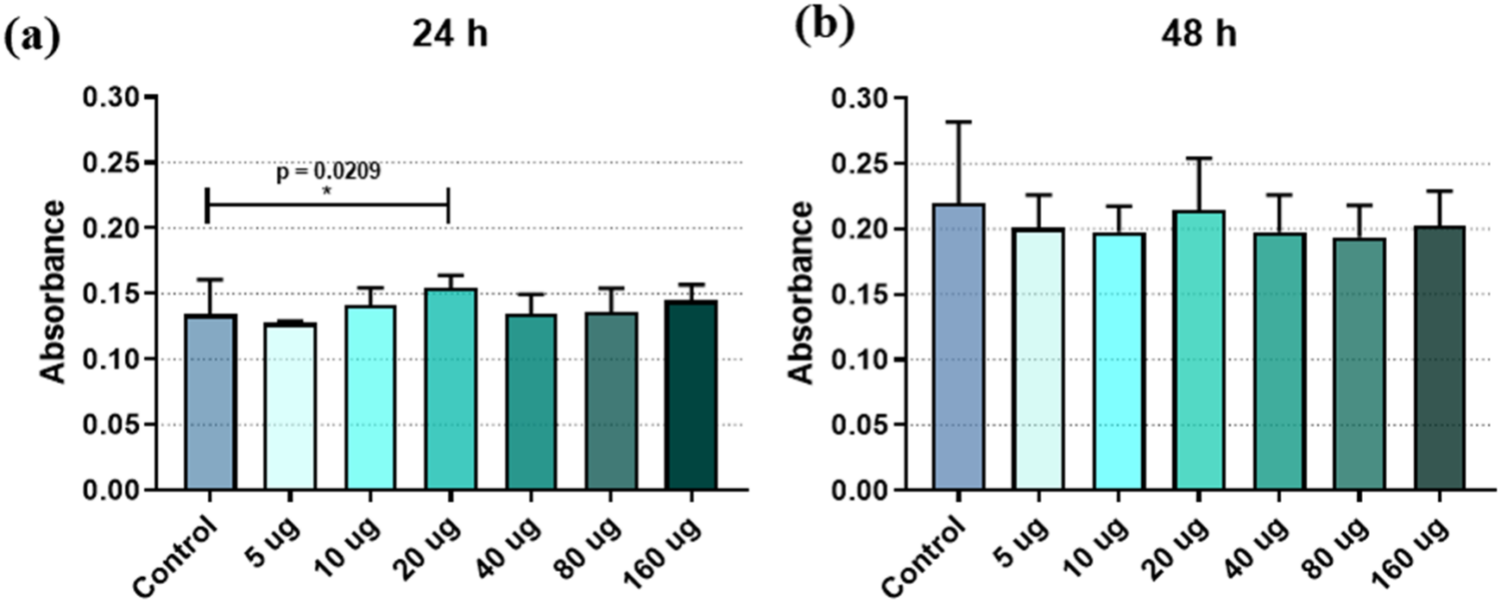
*In vitro* cytotoxicity of S4_SCG-CD evaluated by the MTT assay using 3T3 fibroblast cells after incubation at different S4_SCG-CD concentrations for (a) 24 h and (b) 48 h. A significant variation was observed at 20 μg/L after 24 h (*p* = 0.0209; control: 0.134 ± 0.0088, *n* = 9; treated: 0.1544 ± 0.0036, *n* = 7). Values are expressed as mean ± SEM.

#### 3.5.2 *In Vivo* Testing: Mice

Acute oral toxicity was assessed according to OECD 423,^58^ inspired by prior findings of variable toxicity among nanomaterials.^26^ No adverse behavioral effects were observed at 300 or 2000 mg/kg, during the 24 h and 14-day observation period (Table S9).^59^ Body weight increased postadministration, with only slight, nondose-dependent reductions in the 300 mg/kg group on days 9 (*p* = 0.5441; control: 33.33 ± 1.33; [300]: 32.50 ± 1.33) and 11 (*p* = 0.3253; control: 33.83 ± 1.13; [300]: 32.67 ± 1.13) (Figure 6a,b).^60,61^ No alterations were found in estrous cycle profiles, indicating no reproductive or hormonal disruption (Figure 6c).^62^ Organ-to-body weight ratios showed no significant deviations from controls (Figure 6d), and blood biochemical parameters remained within normal ranges (Table S10), indicating no internal injury or infection. The histology of the liver and kidneys showed preserved architecture without necrosis or inflammation (Figure 6).^59,61^ These results classify S4_SCG-CD as GHS Category 5 with estimated LD_50_ ≥ 5000 mg/kg, indicating low acute toxicity, reinforcing its safety for biomedical and environmental applications.^38,63,64^

**Figure 6:**
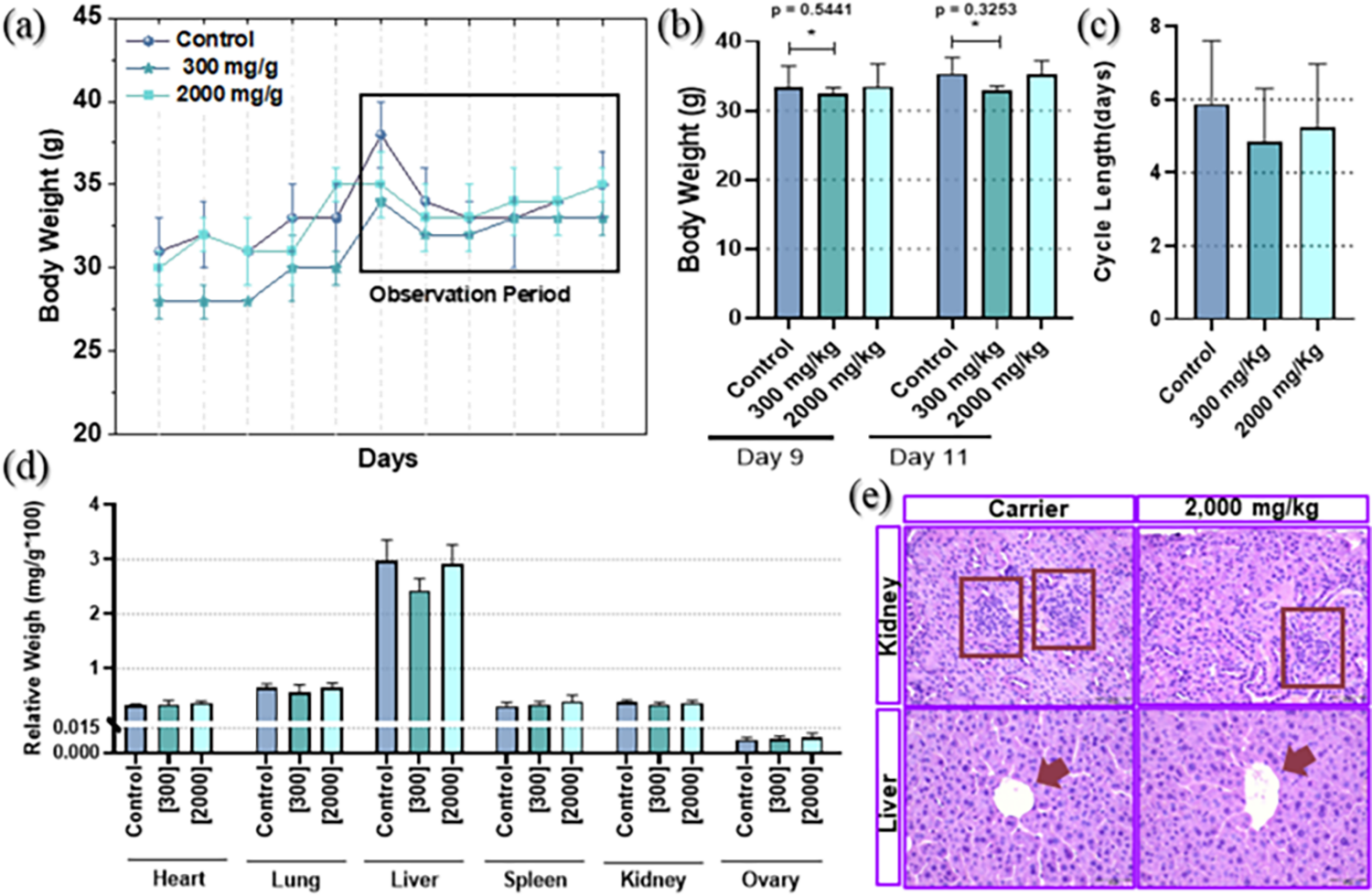
*In vivo* toxicity assessment of S4_SCG-CD in mice. (a) Body weight before and after administration (observation period); (b) statistical comparison of body weight on days 9 (control: 33.33 ± 1.33; treated: 32.50 ± 1.33; *p* = 0.5441) and 11 (control: 33.83 ± 1.13; treated: 32.67 ± 1.13; *p* = 0.3253) in the 300 mg/kg group. Values are expressed as mean ± SEM; (c) estrous cycle dynamics for animals treated with S4_SCG-CD at 300 and 2000 mg/kg; (d) organ-to-body weight ratios for the same doses; (e) histological analysis of kidney and liver from control and S4_SCG-CD -treated mice (2000 mg/kg) with no significant differences.

#### 3.5.3 *In Vivo* Testing: Zebrafish Embryos Test

S4_SCG-CD was further evaluated using zebrafish embryos.^27,65,66^ Survival exceeded 95% at ≤150 μg/L (Figure 7), whereas 500 μg/L caused significant teratogenic effects (*p* = 0.01; control: 98.33 ± 2.89; 500 μg/L: 51.67 ± 2.89), including pericardial and yolk sac edema and tail deformities. Embryos exposed to higher concentrations showed a lower hatching rate at 48 h, as well as increased coagulation, consistent with previously reported embryotoxicity of carbon quantum dots.^67–70^ Although the present study focused on survival and hatching rate as primary end points, assessing additional developmental and physiological parameters, such as heart rate and body length, should be considered in the *in vivo* toxicological evaluation of CD.^27^ Nevertheless, the marked effects observed on survival and hatching, which are robust and widely accepted indicators of embryotoxicity,^70^ were sufficient to support our conclusions. Despite these effects at high doses, S4_SCG-CD was effectively nontoxic at ≤150 μg/L, with an estimated LC_50_ > 150 μg/mL,^27,71,72^ supporting its safe use in biological and environmental applications.

**Figure 7:**
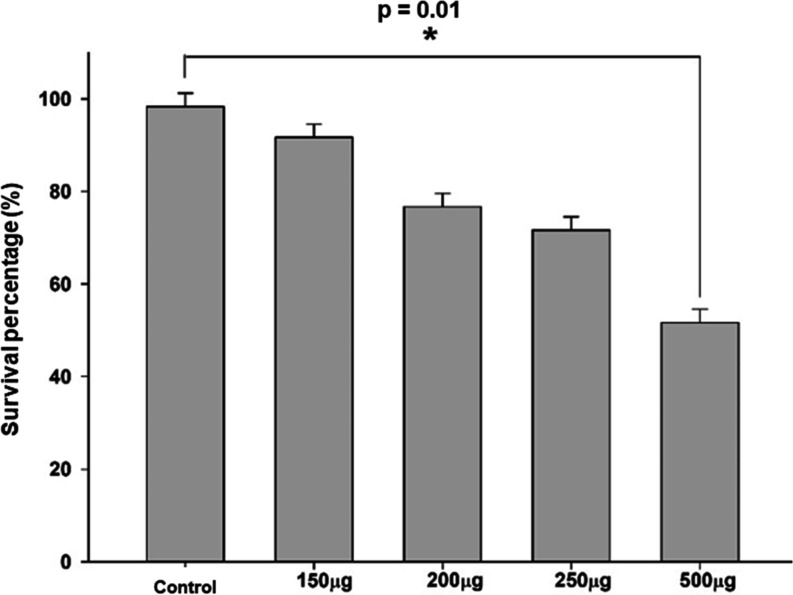
Assessment of acute toxicity in zebrafish embryos. Bar graph showing embryo survival (%) after exposure to S4_SCG-CD at 0 (control), 150, 200, 250, and 500 μg/L. A significant reduction in survival was observed at 500 μg/L compared with the control (*p* = 0.01; control: 98.33 ± 2.89; 500 μg/L: 51.67 ± 2.89). Values are expressed as mean ± SEM.

## 4 Conclusions

Spent coffee grounds were used as a sustainable precursor to synthesize carbon dots (CDs) via pyrolysis under oxidizing conditions. DL-tartaric acid was used as a reference precursor. The design of experiments (DOE) helped optimize the synthesis parameters, including temperature (300 °C, 350 and 400 °C) and residence time (1, 2, and 3 h), guided by thermogravimetric analysis. The synthesized CDs exhibited blue luminescence with excitation-dependent emission. At 310 nm excitation, the highest PLQY were 8,3% for S9_TA-CD (400 °C, 2 h) and 6,0% for S4_SCG-CD (400 °C, 3 h). Optical, FTIR, XRD, and EDS analyses confirmed uniform surface functional groups, semicrystalline structure, and expected elemental composition. Both CD types were pH-responsive, with the S4_SCG-CD sample exhibiting more pronounced spectral changes. *In vitro* and *in vivo* tests demonstrated high biocompatibility and low toxicity, thereby classifying S4_SCG-CD as GHS Category 5. These findings highlight the potential of upcycled coffee waste CDs as biocompatible, photoluminescent nanomaterials for bioimaging, labeling, and biosensing.

## Supplementary Material



## Data Availability

The data supporting this study are available in the manuscript and the Supporting Information. Additional data are available from the corresponding author upon reasonable request.

## Notes

Thermogravimetric and DTA analysis (Figure S1), isothermograms (Figure S2), photoluminescence spectra at different synthesis temperatures of TA and SCG (Figure S2–S3), photoluminescence spectra of TA-CDs as a function of excitation wavelength (310 and 320 nm) and synthesis temperature (300, 350, and 400 h) (Figure S5), photoluminescence spectra of TA-CDs as a function of excitation wavelength (310 and 320 nm) and synthesis time (1, 2, and 3 h) (Figure S6), photoluminescence spectra of SCG-CDs as a function of excitation wavelength (310, 320, 350, and 360 nm) and temperature (300, 350, and 400 °C) (Figure S7), photoluminescence spectra of SCG-CDs as a function of excitation wavelength (310 and 320 nm) and synthesis time (1, 2, and 3 h) (Figure S8), TA-CDs photoluminescence quantum yield (Figure S9), SCG-CDs photoluminescence quantum yield (Figure S10), excitation-dependent photoluminescence spectra (Figure S11), time-resolved photoluminescence spectra (Figure S12), S4_SCG-CD EDS spectroscopy (Figure S13), and S4_SCG-CD diffractogram pattern (Figure S14); tables of photoluminescence data and extended toxicity results in mice and zebrafish (PDF)
