# Supplementary material for: Fluorescent Carbon Dots from Biomass Waste: Photoluminescent Behavior and Toxicity Profile Validated In Vitro and In Vivo
Source: ACS Omega. 2026 May 22;11(22):32194–204. doi: 10.1021/acsomega.5c12878 (PMC13261416; doi:10.1021/acsomega.5c12878)
Supplement: Supplementary file 1 [file ao5c12878_si_001.pdf]

Supporting Information

Fluorescent Carbon Dots from Biomass Waste: Photoluminescent Behavior and Toxicity Profile Validated In Vitro and In Vivo

*Luana Caroline de Oliveira Lima[1], Janaina Domingas Alves[2], Amanda Maria Siqueira Moreira[3], Erika Cristina Jorge[3], Hélio Batista dos Santos[4], Ralph Gruppi Thomé[4], Paulo Henrique Almeida Campos-Junior[2], Marco Antônio Schiavon[1]**

[1]Grupo de Pesquisa em Química de Materiais (GPQM), Departamento de Ciências Naturais, Universidade Federal de São João del-Rei, São João del-Rei, 36.301-160, Brazil.

[2]Laboratório de Pesquisa em Reprodução (LAPER), Departamento de Ciências Naturais, Universidade Federal de São João del-Rei, São João del-Rei, 36.301-160, Brazil.

[3]Laboratório de Biologia Oral e do Desenvolvimento (LABODE), Departamento de Morfologia, Universidade Federal de Minas Gerais, Belo Horizonte, 31.270-901, Brazil.

[4] Laboratório de Processamento de Tecidos, Campus Centro-Oeste Dona Lindu, Universidade Federal de São João del-Rei, Divinópolis, 35.501-296, Brazil.

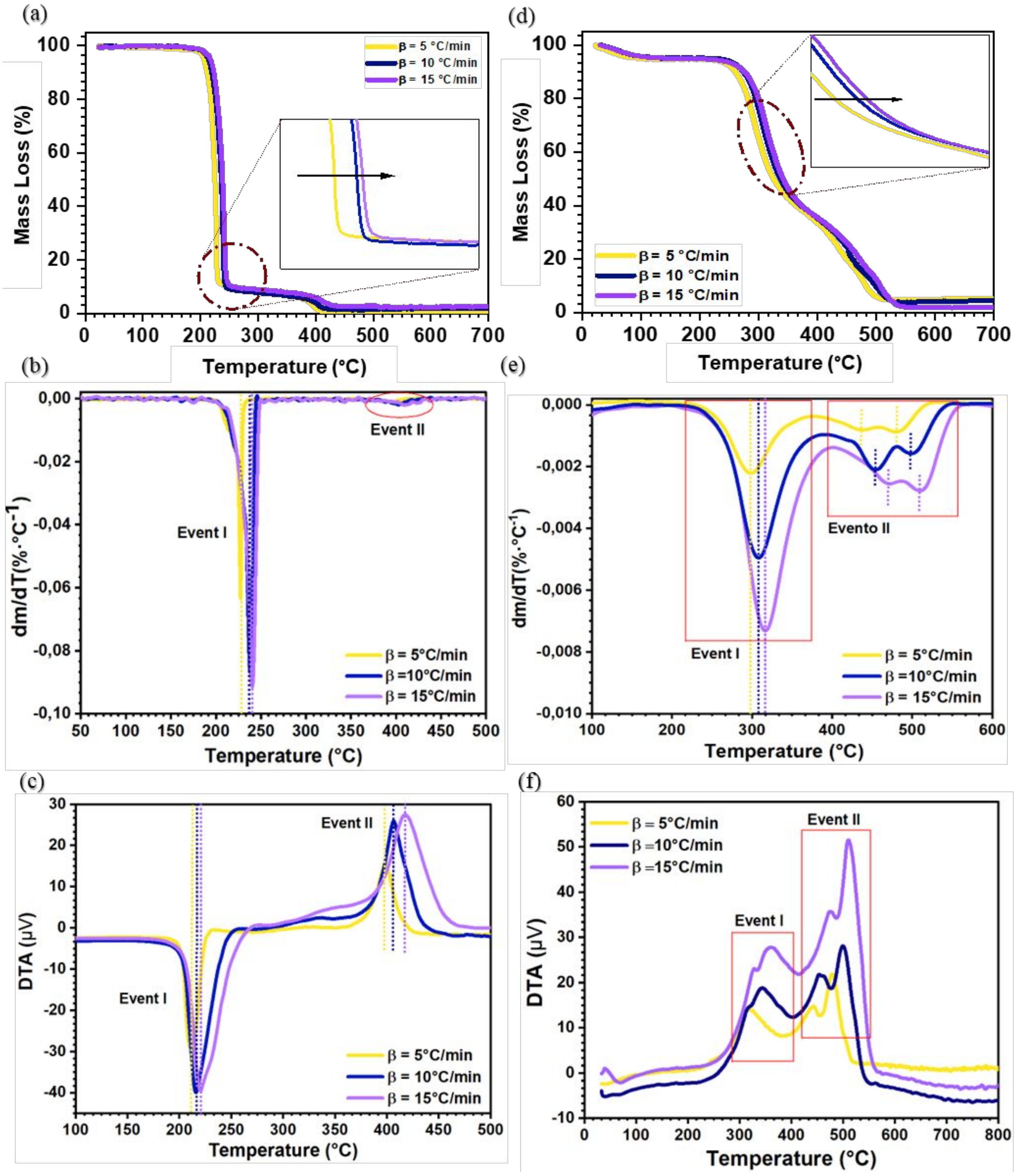

Figure S1. Thermogravimetric and thermal analyses of TA and SCG performed at different heating rates (β = 5, 10, and 15 °C min⁻¹). Panels (a) and (d) show the TGA thermograms of TA and SCG, respectively, obtained at varying heating rates; the insets highlight the systematic shift of the mass-loss profiles toward higher temperatures with increasing β. Panels (b) and (e) present the corresponding DTG curves. For TA, two main thermal events are observed, with the first being more pronounced, whereas SCG exhibits three thermal events that become progressively better defined as the heating rate increases, with β = 10 °C min⁻¹ showing the clearest separation. Panels (c) and (f) display the DTA curves of TA and SCG, respectively, enabling the distinction between endothermic and exothermic processes associated with the observed thermal events.

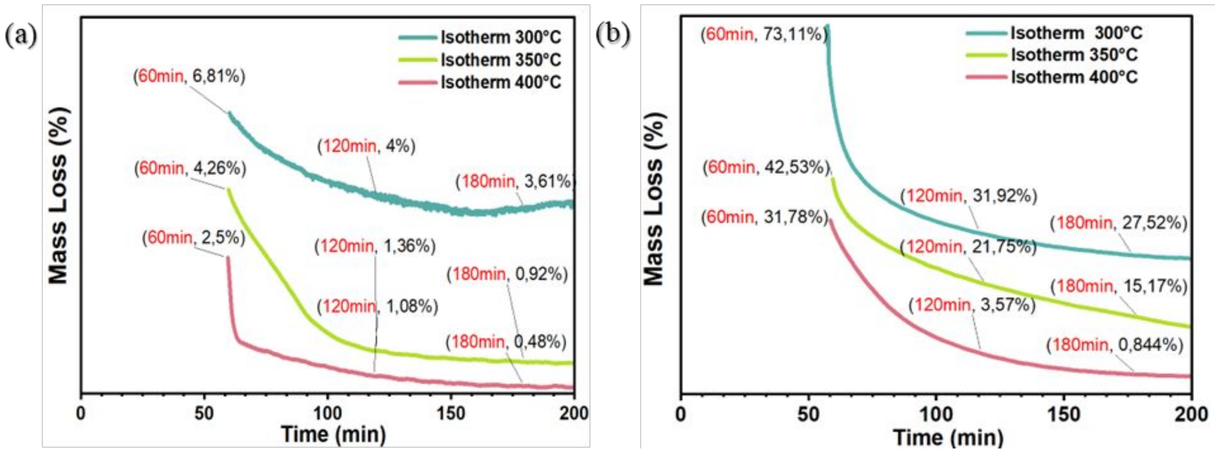

Figure S2. Isothermal TGA profiles 300, 350, and 400 °C for (a) DL-tartaric acid and (b) spent coffee grounds. The isothermal dwell times of 60, 120, and 180 min are indicated in red, while the corresponding percentages of remaining mass at each time are reported in black.

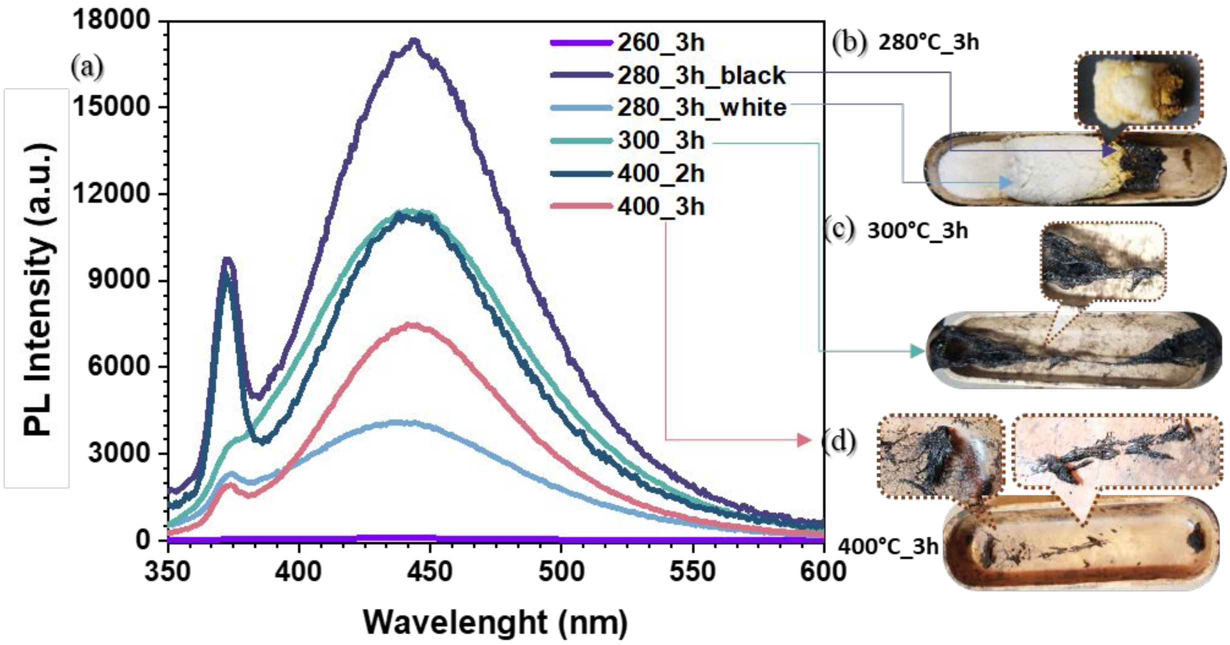

Figure S3. (a) Photoluminescence (PL) screening of DL-tartaric acid (TA)–derived samples synthesized performed over the temperature range of 260 to 400 °C at different residence times; (b-d) Photographs of TA samples synthesized at fixed conditions of (b) 280 °C for 3 h, (c) 300 °C for 3 h, and (d) 400 °C for 3 h, illustrating sample's aspects.

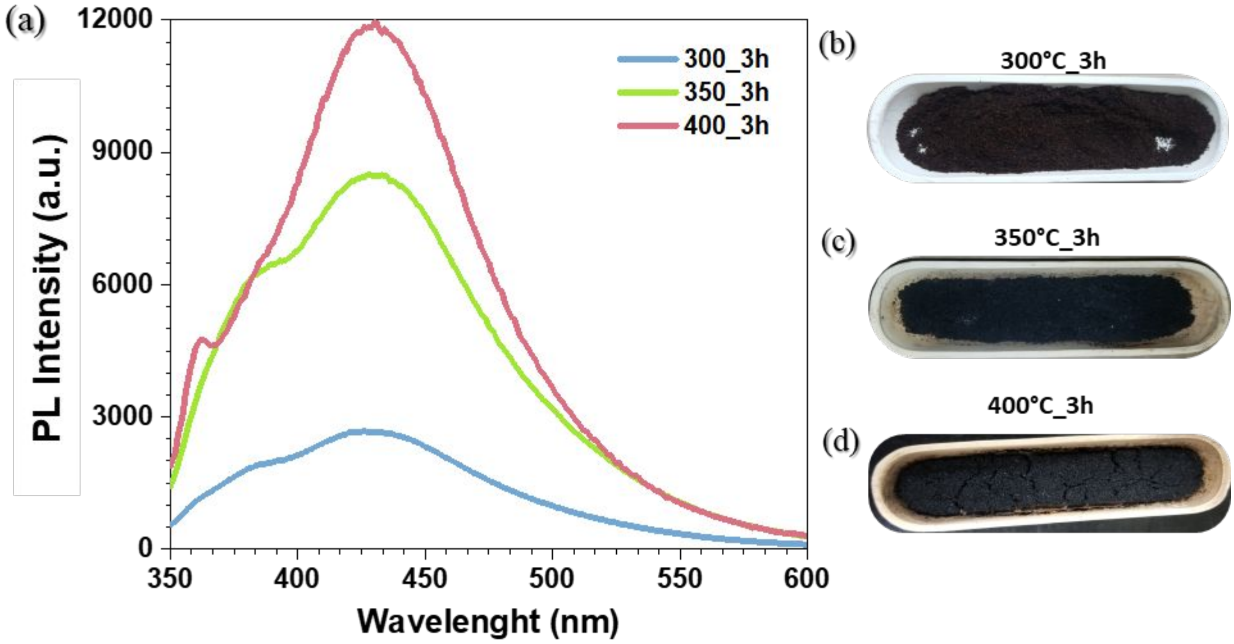

Figure S4. (a) Photoluminescence (PL) screening of Spent Coffee Grounds (SCG)–derived samples synthesized performed over the temperature range of 300 to 400 °C at 3h each; (b–d) Photographs of SCG samples synthesized at fixed conditions of (b) 300 °C for 3 h, (c) 350 °C for 3 h, and (d) 400 °C for 3 h, illustrating sample's aspects.

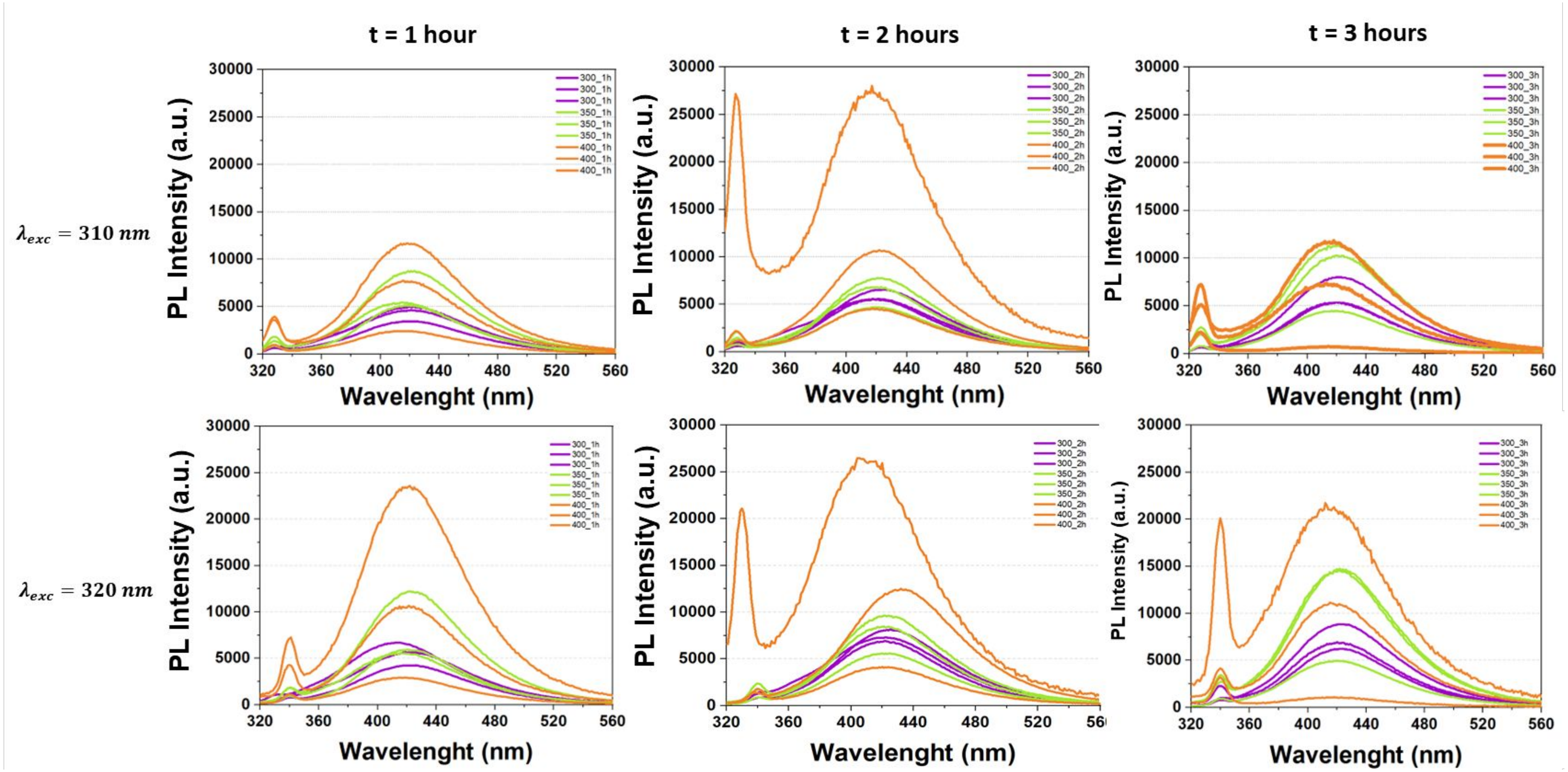

Figure S5. Photoluminescence spectra of TA-CDs as a function of excitation wavelength (310 and 320 nm) and synthesis temperature (300, 350 and 400 °C). Spectra at each temperature and time were measured in triplicate. Colors represent different synthesis temperatures: purple – 300 °C, green – 350 °C, and orange – 400 °C.

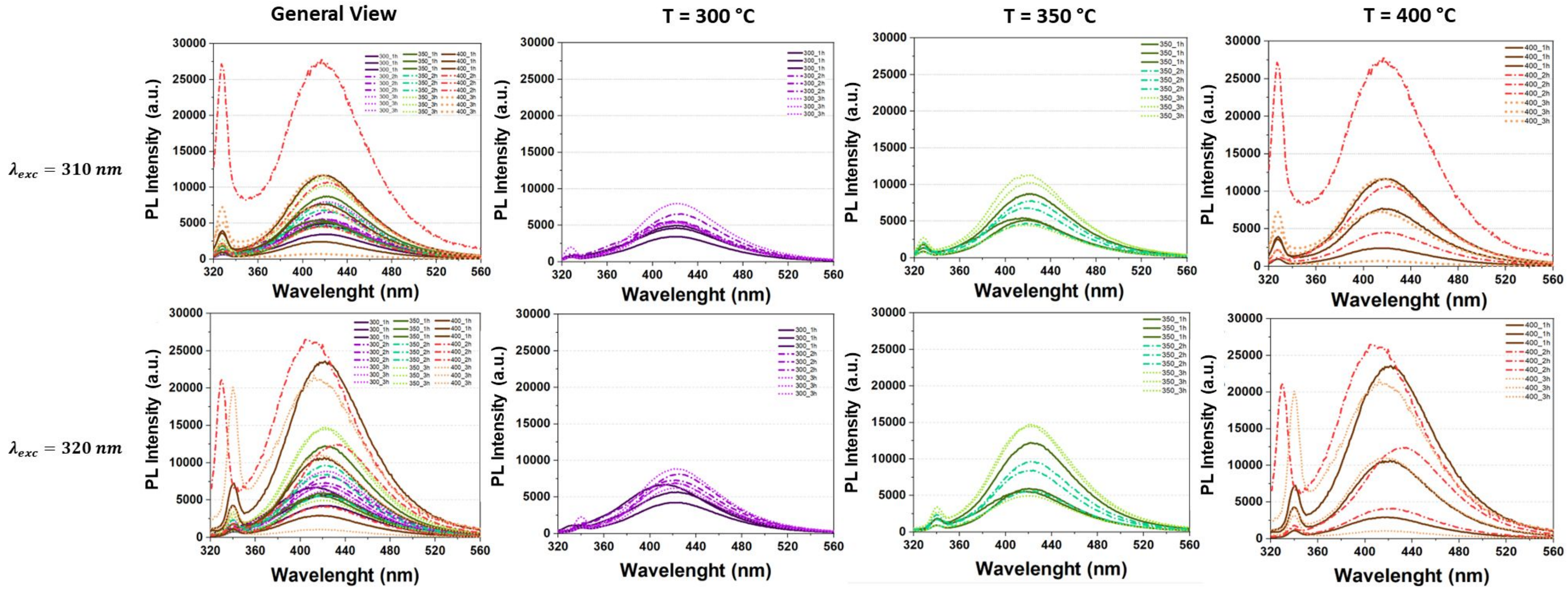

Figure S6. Photoluminescence spectra of TA-CDs as a function of excitation wavelength (310 and 320 nm) and synthesis time (1 h, 2 h and 3 h). Spectra at each time were measured in triplicate. Line styles represent different synthesis times: solid line – 1 h, dash-dot line – 2 h, and dotted line – 3 h.)

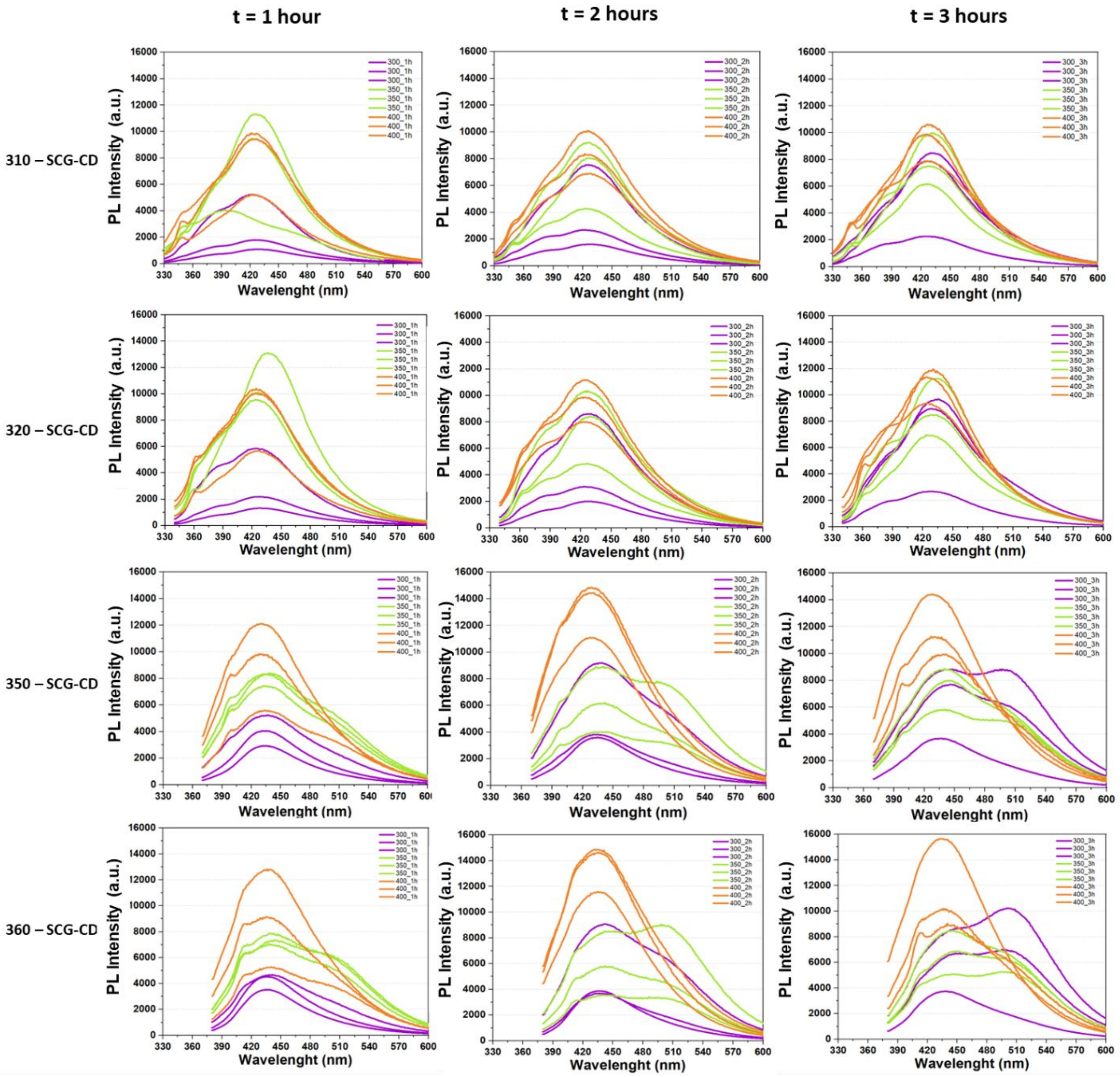

Figure S7. Photoluminescence spectra of SCG-CDs as a function of excitation wavelength (310, 320, 350 and 360 nm) and temperature (300, 350 and 400 °C). Spectra at each temperature and time were measured in triplicate. Colors represent different synthesis temperatures: purple – 300 °C, green – 350 °C, and orange – 400 °C.

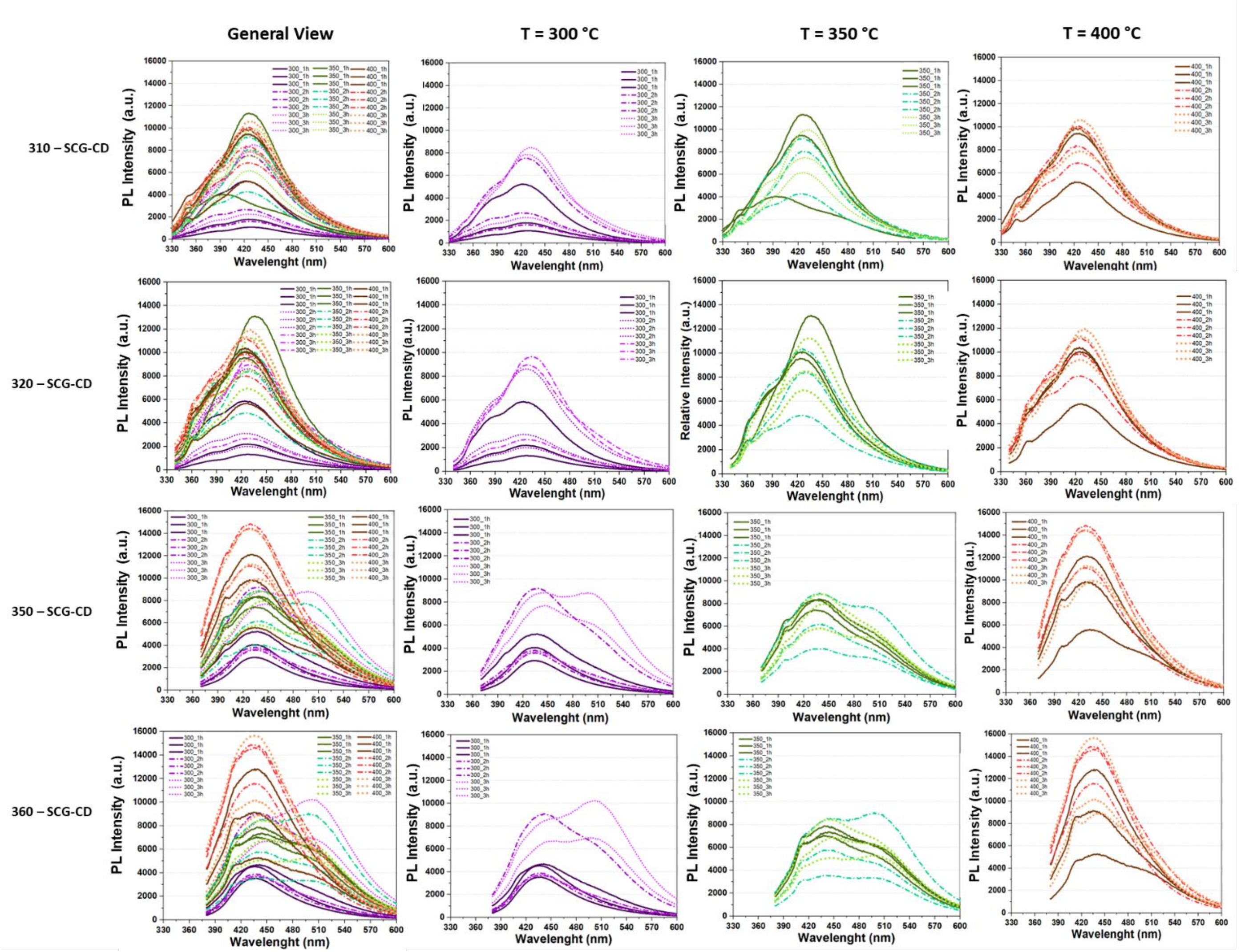

Figure S8. Photoluminescence spectra of SCG-CDs as a function of excitation wavelength (310 and 320 nm) and synthesis time (1 h, 2 h and 3 h). Spectra at each time were measured in triplicate. Line styles represent different synthesis times: solid line – 1 h, dash-dot line – 2 h, and dotted line – 3 h.)

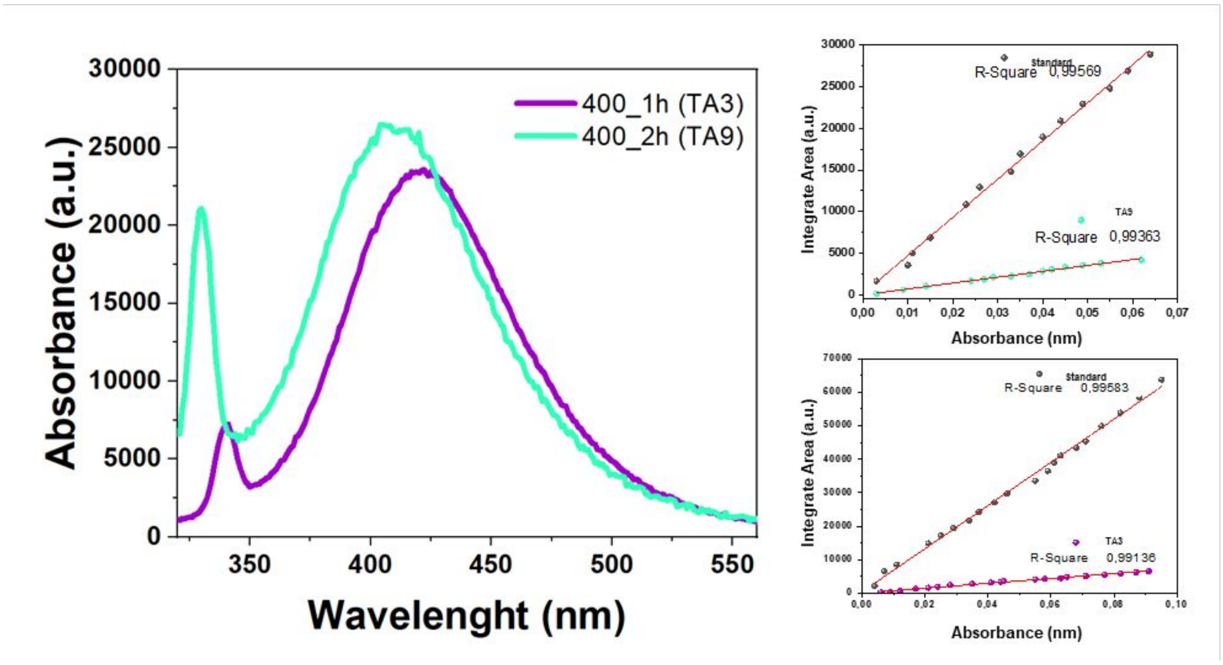

Figure S9. TA-CDs Photoluminescence quantum yield (PLQY): TA9 (400 °C, 2 h) and T3 (400 °C, 1 h), at λ_exc = 310 nm.

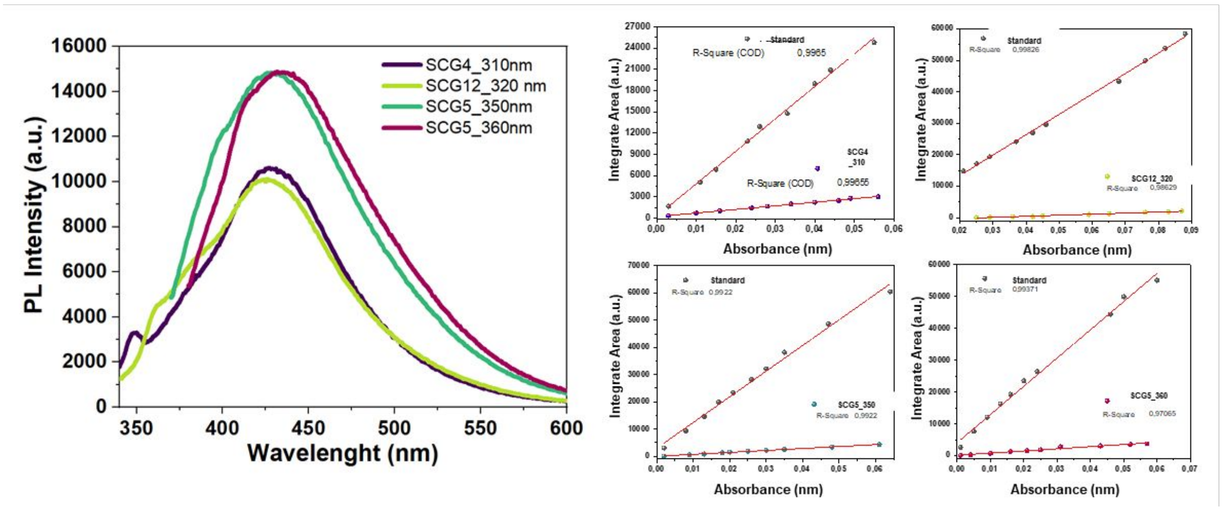

Figure S10. SCG-CDs photoluminescence quantum yield (PLQY): SCG4 (λ_exc = 310 nm), SCG12 (λ_exc = 320 nm), SCG5 (λ_exc = 350 nm), SCG5 (λ_exc = 360 nm).

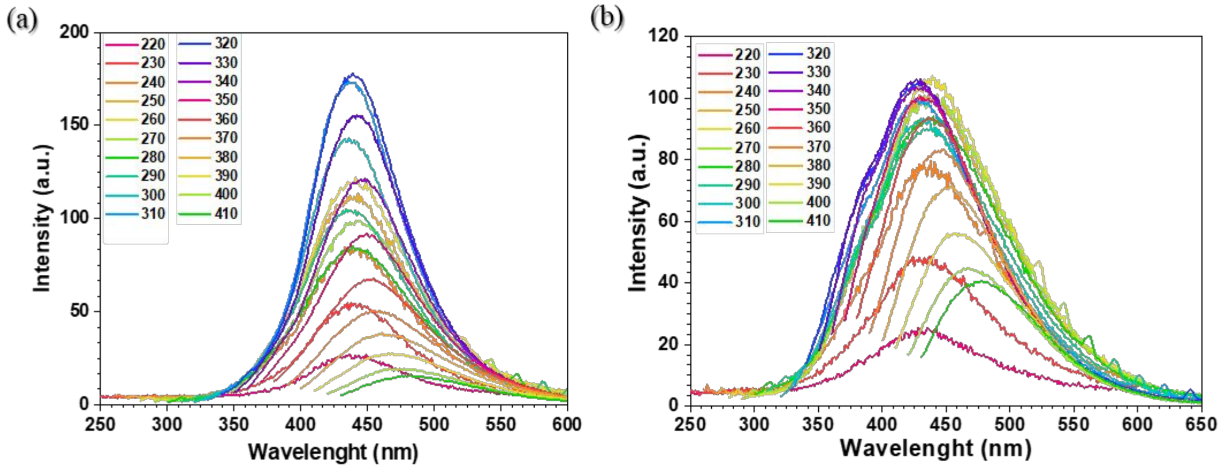

Figure S11. Excitation-dependent photoluminescence of (a) S9_TA-CD and (b) S4_SCG-CD.

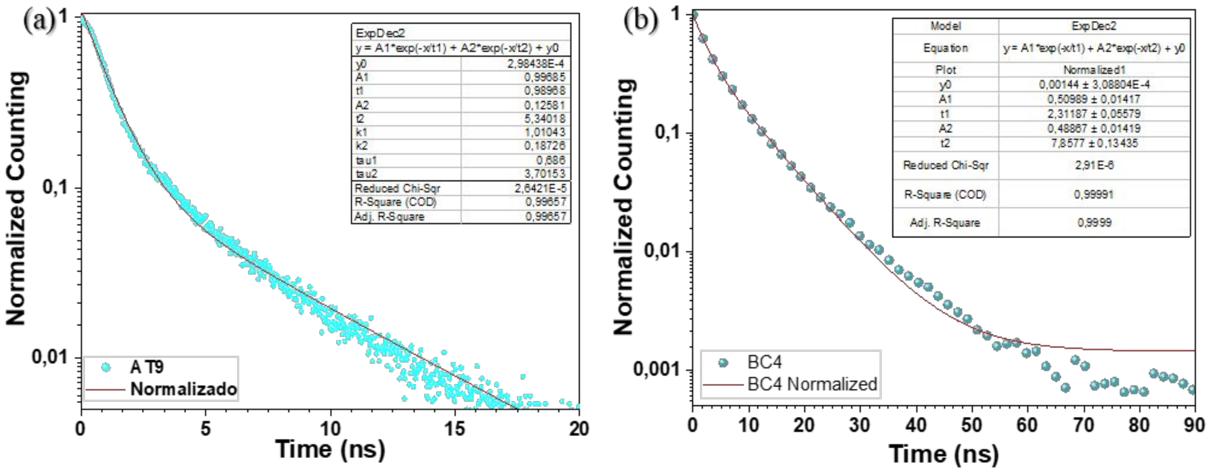

Figure S12. Time-resolved photoluminescence spectra of (a) S9_TA-CD and (b) S4_SCG-CD.

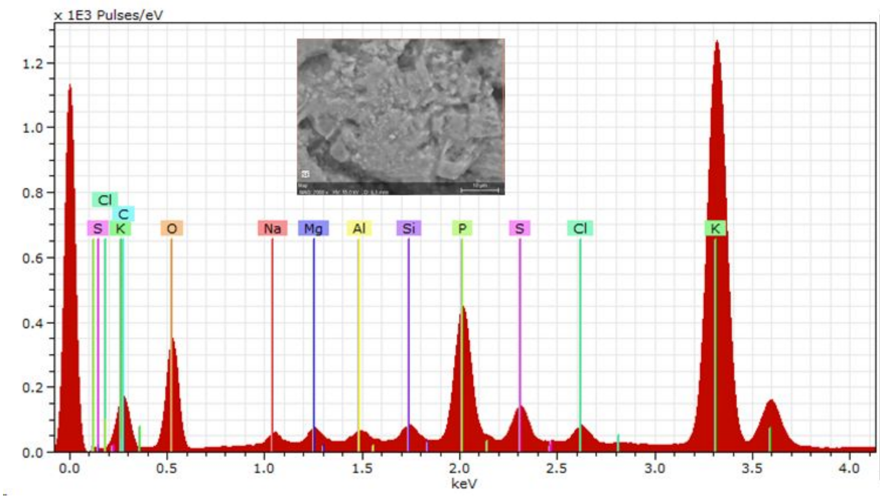

El	AN	Series	norm. C [wt.%]	Atom. C [at.%]
K	19	K-series	39.05	21.00
O	8	K-series	38.02	49.97
C	6	K-series	12.46	21.81
S	16	K-series	4.45	2.92
P	15	K-series	3.55	2.41
Al	13	K-series	0.81	0.63
Cl	17	K-series	0.77	0.46
Na	11	K-series	0.63	0.57
Mg	12	K-series	0.23	0.20
Si	14	K-series	0.03	0.02
		Total:	100.00	100.00

Figure S13. Distribution of elements via electron scattering spectroscopy coupled with scanning electron microscopy, and distribution table showing the elemental percentage of each element found in S4_SCG-CD.

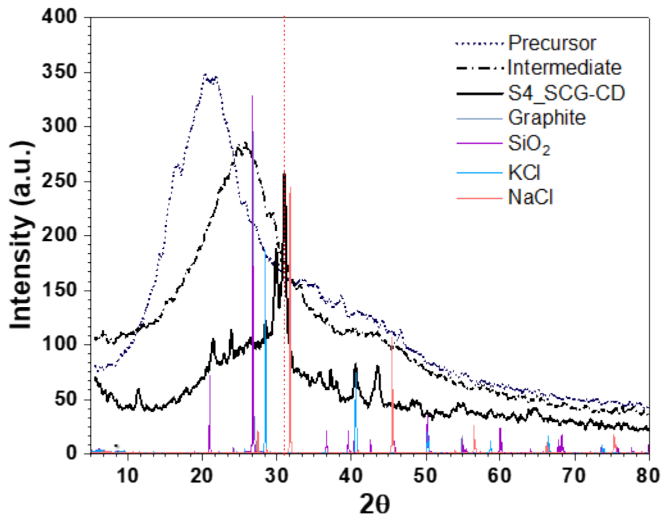

Figure S14. Diffractogram pattern of spent coffee grounds (precursor), the intermediate (solid material that is discarded after filtration) – and S4_SCG-CD, traversed between angles of 5°–60° at a step of 2 °/s. Graphite, SiO2, KCl, and NaCl patterns obtained from the RUFF XRD data network.

TABLES

Table S1. Summary of best conditions and PLQY values for TA-CD and SCG-CD.

Precursor	Sample ID	λ_{exc}	PLQY
Tartaric Acid Carbon Dots	400°C_1h (AT3)	310 nm	6.1%
	400°C_2h (AT9)	310 nm	8.3%
Spent Coffee Ground Carbon Dots	400°C_3h (BC4)	310 nm	6.0%
	350°C_1h (BC12)	320 nm	2.8%
	400°C_2h (BC15)	350 nm	4.1%
	400°C_2h (BC15)	360 nm	4.1%

Table S2. Statistical analysis (ANOVA) of the influence of factors (temperature and time) on the properties of TA-CDs.

Source	GL	SQ (Aj.)	QM (Aj.)	Value F	P–Value
Model	8	$4.76019 x 10^{11}$	59502363127	1.02	0.458
Linear	4	$2.34645 x 10^{11}$	58661258965	1.00	0.432
Temperature	2	$1.43857 x 10^{11}$	71928615015	1.23	0.316
Time	2	90787805830	45393902915	0.78	0.475
2-factors interactions	4	$2.41374 x 10^{11}$	60343467290	1.03	0.418
Temperature*time	4	$2.41374 x 10^{12}$	60343467290	1.03	0.418
Error	18	$1.05290 x 10^{12}$	58494634285		
Total	26	$1.52892 x 10^{12}$			

Table S3. Statistical analysis (ANOVA) of the influence of terms on the properties of TA-CDs.

Term	Coef.	EP de Coef.	T – value.	P–Value	VIF
Constant	385165	46545	8.28	0.000	
Temperature					
300	-94071	65825	-1.43	0.170	1.33
350	10224	65825	0.16	0.878	1.33
Time					
1	-68887	65825	-1.05	0.309	1.33
2	72976	65825	1.11	0.282	1.33
Temperature*Time					
300 1	-5728	93091	-0.06	0.952	1.78
300 2	-57083	93091	-0.61	0.547	1.78
350 1	16292	93091	0.18	0.863	1.78
350 2	-109571	93091	-1.18	0.255	1.78

Table S4. Statistical analysis (ANOVA) of the influence of factors (temperature and time) on the properties of SCG-CDs.

Source	GL	SQ (Aj.)	QM (Aj.)	F Value	P–Value
Model	8	$1.34846 x10^{12}$	$1.68557 x10^{11}$	2.51	0.050
Linear	4	$1.21782 x 10^{12}$	$3.04455 x10^{11}$	4.53	0.010
Temperature	2	$1.08099 x1 0^{12}$	$5.40493 x10^{11}$	8.04	0.003
Time	2	$1.36833 x10^{11}$	68416729960	1.02	0.381
2-factors interactions	4	$1.30638 x10^{11}$	32659525565	0.49	0.746
Temperature*time	4	$1.30638 x10^{11}$	32659525565	0.49	0.746
Error	18	$1.20960 x10^{12}$	67199988469		
Total	26	$2.55806 x10^{12}$			

Table S5. Statistical analysis (ANOVA) of the influence of terms on the properties of SCG-CDs.

Term	Coef.	EP de Coef.	T – value.	P–Value	VIF
Constant	730667	49889	14.65	0.000	
Temperature					
300	-271496	70553	-3.85	0.001	1.33
350	66667	70553	0.94	0.357	1.33
Time					
1	-70873	70553	-1.00	0.328	1.33
2	-26488	70553	-0.38	0.712	1.33
Temperature*Time					
300 1	-103862	99778	-1.04	0.312	1.78
300 2	-6667	99778	-0.07	0.947	1.78
350 1	106616	99778	1.07	0.299	1.78
350 2	-24746	99778	-0.25	0.807	1.78

Table S6. Summary of parameters obtained from time-resolved photoluminescence spectra and calculated values of average lifetime, radiative and non-radiative events for Tartaric Acid and Spent Coffee Grounds.

Sample	S9_TA-CD	S4_SCG-CD
α_1	0.99	0.50
τ_1 (ns)	0.68	1.60
α_2	0.12	0.48
τ_2 (ns)	3.70	5.44
k_r (ns)	0.04	0.01
k_{nr} (ns)	0.48	0.20
QY(%)	8.00	6.00
<t>	1.90	4.54

Table S7. Main FTIR absorption bands identified for coffee grounds and the corresponding to S4_SCG-CD.

Wavenumber range (cm^{-1})	Functional group assignment	Observed in	Notes
3650 – 3100	O–H (aromatic or phenolic structure)	SCG	Broad stretching, indicative of aromatic or phenolic OH groups.
~3080	=C–H (olefins)	SCG	Alkenyl C–H stretching; signal confirmed by harmonic in 1680–1630 cm^{-1}.
1820 – 1760	Acyl halide C=O	SCG	Sharp stretching band in carbonyl region.
1820 – 1630	Ester C=O	SCG / S4_SCG-CD	Strong stretching from ester groups.
1700 – 1630	Aryl ketone / amide C=O	SCG / S4_SCG-CD	Present in complex organic structures.
1644	C=O / C=N (amide-related)	S4_SCG-CD	Nitrogen-containing amide or imine group.
1570 – 1500; 1380 – 1300	Nitro group ($–NO_2$)	SCG	Symmetric and asymmetric stretching of NO_2.
1527	O=C−NH / C=C (amide or conjugated C=C)	S4_SCG-CD	Associated with amide or aromatic C=C bonds.

Table S8. Main FTIR absorption bands identified for tartaric acid and the corresponding S9_TA-CD

Wavenumber range (cm^{-1})	Functional group assignment	Observed in	Notes
3650 – 3100	O–H (alcohol / carboxylic acid)	TA / AT9	Broad band, more intense and overlapped in S9_TA-CD.
~3080	=C–H (olefinic/aromatic)	AT9	Suggests presence of unsaturated/aromatic systems.
3000 – 2840	C–H (alkanes)	TA / S9_TA-CD	Typical aliphatic C–H stretching.
2830 – 2700	C–H (aldehyde)	TA	Weak C–H stretching of aldehyde groups.
1820 – 1630	C=O (carboxylic acid)	TA / S9_TA-CD	Strong absorption due to carbonyl groups.
1591	Conjugated C=C / aromatic ring	S9_TA-CD	Suggests formation of conjugated carbon domains.
1465	C–H bending (alkane harmonic)	TA / S9_TA-CD	Aliphatic group vibration mode.
1150	C–OH (tertiary alcohol)	S9_TA-CD	Broad O–H stretching of tertiary alcohols.

Table S9. Behavioral patterns of mice in the control group at concentrations of 300 and 2000 mg/kg during the 24-h period of S4_SCG-CD

	Observation period	5 min			10 min			20 min			30 min			60 min			2 h			4 h			6 h			12 h			24 h		
	Concentration (mg/kg)	Control	300	2000	Control	300	2000	Control	300	2000	Control	300	2000	Control	300	2000	Control	300	2000	Control	300	2000	Control	300	2000	Control	300	2000	Control	300	2000
Parameters	Piloerection	N	N	N	N	N	N	N	N	N	N	N	N	N	N	N	N	N	N	N	N	N	N	N	N	N	N	N	N	N	N
	Corneal dryness	N	N	N	N	N	N	N	N	N	N	N	N	N	N	N	N	N	N	N	N	N	N	N	N	N	N	N	N	N	N
	Change in breathing	N	N	N	N	N	N	N	N	N	N	N	N	N	N	N	N	N	N	N	N	N	N	N	N	N	N	N	N	N	N
	Summative activity	N	N	N	N	N	N	N	N	N	N	N	N	N	N	N	N	N	N	N	N	N	N	N	N	N	N	N	N	N	N
	Seizures	N	N	N	N	N	N	N	N	N	N	N	N	N	N	N	N	N	N	N	N	N	N	N	N	N	N	N	N	N	N
	Itch	N	N	N	N	N	N	N	N	N	N	N	N	N	N	N	N	N	N	N	N	N	N	N	N	N	N	N	N	N	N
	Mortality	N	N	N	N	N	N	N	N	N	N	N	N	N	N	N	N	N	N	N	N	N	N	N	N	N	N	N	N	N	N

Obs.: P., positive; N., negative

Table S10. Hemogram of animals from the control group and the 2000 mg/kg concentration group of S4_SCG-CD

Parameters	Control (n=3)	2000 mg/g (n=3)	Parameters	Control (n=3)	2000 mg/g (n=3)
Global leukocyte (μl)	6666.7 ± 1322.4	12466.7 ± 6489.1	Hematocrit (%)	33.9 ± 2.2	36.1 ± 2.8
Segmented (%)	16.0 ± 5.7	15.3 ± 3.4	VCM (%)	45.4 ± 0.1	45.8 ± 0.3
Lymphocytes (%)	83.3 ± 6.6	82.7 ± 1.9	HCM (%)	17.0 ± 1.6	16.5 ± 0.4
Monocytes (%)	0.7 ± 0.9	2.0 ± 1.6	CHCM (%)	36.3 ± 1.9	36.0 ± 0.7
Red blood cells (%)	7.3 ± 0.7	7.9 ± 0.6	RDW (%)	15.2 ± 1.3	13.4 ± 0.7
Hemoglobin (%)	12.3 ± 0.4	13.0 ± 1.1	Platelets (%)	131500.0 ± 32500.0	185333.3 ± 87263.3

EQUATIONS

Calculation of Fluorescence Quantum Yields from Acquired Data:

The photoluminescence quantum yield (PLQY) of the nanomaterials was determined using the comparative method of Williams (Equation 1)

$$\Phi_x = \Phi_{ST}\left(\frac{Grad_X}{Grad_{ST}}\right)\left(\frac{\eta_X^2}{\eta_{ST}^2}\right) \qquad \text{Equation 1}$$

Where the subscripts ST and X denote standard and test respectively, Φ is the fluorescence quantum yield, Grad the gradient from the plot of integrated fluorescence intensity vs absorbance, and η the refractive index of the solvent

Ultrapure water (Milli-Q, refractive index η = 1.33) was used as the solvent for sample dispersions, and quinine sulfate, dispersed in 0.1 M H_2SO_4 (η = 1.33) with a Φ_f of 54% at the same λ_ex as the sample, was employed as the reference standard. The relationship between the integrated photoluminescence intensity and absorption was obtained by plotting a calibration curve and fitting a linear regression.

S9_TA-CD – Regression Analysis:

$$\begin{aligned}\text{Intensidade 310 nm} &= 385165 - 94071\,A + 10224\,B + 83847\,C - 68887\,1H \\ &+ 72976\,2H - 4089\,3H - 5728\,A*1H - 57083\,A*2H \\ &+ 62811\,A*3H + 16292\,B*1H - 109571\,B*2H \\ &+ 93279\,\mathrm{B}*3H - 10564\,C*1\mathrm{H} + 166654\,\mathrm{C}*2H \\ &- 156090\,C*3H\end{aligned} \qquad \text{Equation 2}$$

S4_SCG-CD – Regression Analysis:

$$\begin{aligned}Intensidade\ 310 &= 730667 - 271496\,A + 66667\,B + 204829\,C - 70873\,1H \\ &- 26488\,2H + 97361\,3H - 103862\,A*1H - 6667\,A*2H \\ &+ 110528\,A*3H + 106616\,B*1H - 24746\,B*2H \\ &- 81871\,B*3H - 2754\,C*1H + 31412\,C*2H - 28658\,C \\ &*3H\end{aligned} \qquad \text{Equation 3}$$

Biexponential decay from time-resolved photoluminescence analysis:

$$y(t) = \alpha_1 \exp\left(-\frac{t}{\tau_1}\right) + \alpha_2 \exp\left(-\frac{t}{\tau_2}\right) \qquad \text{Equation 4}$$
